# Ensemble approach to predict specificity determinants: benchmarking and validation

**DOI:** 10.1186/1471-2105-10-207

**Published:** 2009-07-02

**Authors:** Saikat Chakrabarti, Anna R Panchenko

**Affiliations:** 1 National Center for Biotechnology Information, National Library of Medicine, National Institutes of Health, Bethesda, Maryland, USA

## Abstract

**Background:**

It is extremely important and challenging to identify the sites that are responsible for functional specification or diversification in protein families. In this study, a rigorous comparative benchmarking protocol was employed to provide a reliable evaluation of methods which predict the specificity determining sites. Subsequently, three best performing methods were applied to identify new potential specificity determining sites through ensemble approach and common agreement of their prediction results.

**Results:**

It was shown that the analysis of structural characteristics of predicted specificity determining sites might provide the means to validate their prediction accuracy. For example, we found that for smaller distances it holds true that the more reliable the prediction method is, the closer predicted specificity determining sites are to each other and to the ligand.

**Conclusion:**

We observed certain similarities of structural features between predicted and actual subsites which might point to their functional relevance. We speculate that majority of the identified potential specificity determining sites might be indirectly involved in specific interactions and could be ideal target for mutagenesis experiments.

## Background

Proteins within a homologous family usually share a 'general' function while functional specificities may vary within a family. Proteins belonging to subgroups (subfamilies) may evolve slightly different functions and different substrate specificities while maintaining an overall function of a family. Positions that are conserved within subsets of closely related proteins in a given family, but are variable between the subsets, are likely to be involved in functional specificity [1-4]. These sites generally determine the protein specificity either by binding to specific substrate or through interaction with specific protein partner. In many cases, comparative techniques allow one to assign common specificity to groups of proteins, and thus provide data for analysis of specificity determining residues in protein sequences [5].

Several computational techniques have been designed to predict specificity determining sites. The evolutionary trace method identified invariant specific residues by partitioning the phylogenetic tree into subgroups of similar sequences [6,7]. Various site-specific conservation scores have been offered to distinguish conserved functionally important sites from the background of neutral changes, such as relative entropy, mutual entropy or "sequence harmony" [8-17]. Other methods have overcome the limitation of requirement of pre-defined subgrouping by simultaneous identification of optimal groups and specific conserved positions [18,19]. In our previous study [20] we reported a method that encodes the specific conservation pattern within and between subfamilies using amino acids' physico-chemical properties and the evolutionary rates.

Despite several recent efforts, successful prediction of specificity determining sites (called "subsites" hereafter) still remains to be a difficult task. On one hand, the lack of success might be caused by the limited availability of experimentally characterized subsites which, in turn, can make the methods to be biased toward the prediction of certain types of subsites. Moreover, the specificity may be affected by subtle changes in residue stereochemistry which can be difficult to detect and in many cases subsites are located on flexible or disordered loops that are difficult to characterize. Therefore, a comparative analysis of subsite prediction methods on a larger, comprehensive dataset will provide a reliable evaluation revealing the weak and strong points of each method. Additionally, a meta-prediction approach combining the results from best performing methods would be also useful for identification of new potential subsites. It should be mentioned that division of protein families into subfamilies is also a crucial step and requires careful manual intervention. However, reasonable success of several recent methods [9,18,21,22] is encouraging to advocate use of automated subgrouping in specificity site prediction.

In this study, prediction performances of almost all available methods were tested and validated using a comprehensive dataset comprising 20 protein families for which experimental data are available for subsites. Several additional potential subsites were also predicted by combining the results of methods showing the best performance on the benchmark of 20 families (using our dataset), SPEER [20] GroupSim [16] and MultiRELIEF [17]. Potential subsites, commonly predicted either by all three best methods (C3 sites) or by any two (C2 sites) can be excellent targets for mutagenesis studies to reveal specificity determining sites. We also showed that the analysis of structural characteristics of actual and predicted subsites might provide the means to validate the prediction accuracy.

## Results

### Performance evaluation of subsite prediction methods

**Figure 1:**
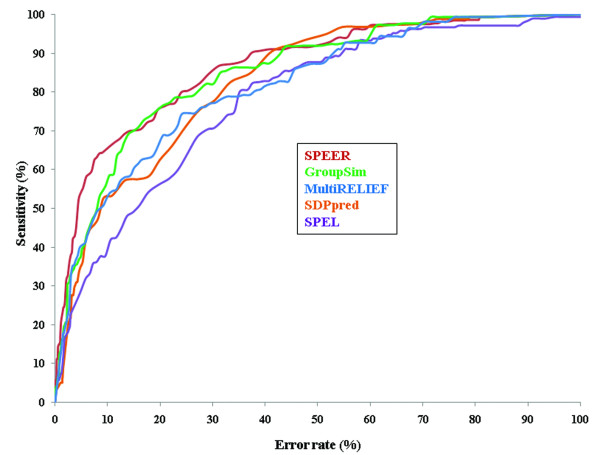
**Comparison of prediction performances**. ROC-curves for prediction of subsites are shown for SPEER, GroupSim, MultiRELIEF, SDPpred and SPEL methods. Sensitivity and error rates are estimated based on the number of true positives (correctly predicted actual subsites) and false positives (predicted sites which are not actual subsites) found at each score cutoff. Sensitivity (TP/TP+FN) is defined as a number of true positives (TP) found at each score threshold divided by the sum of true positives and false negatives (FN), where false negatives are defined as actual subsites below the score threshold. Error rate (FP/FP+TN) is estimated as the number of false positives (FP) divided by the sum of false positives and true negatives (TN, non-subsites below the score threshold).

Multiple sequence alignments of 20 families (validation dataset) were used to identify actual subsites. Experimentally supported subsites (195 actual subsites) from these families were considered as gold standards for the evaluation of performance of five prediction methods, namely SPEER [20] GroupSim [16] and MultiRELIEF [17], SDPpred [12] and SPEL [18]. The prediction sensitivities of these five methods are shown (Figure 1) as Receiver Operating Characteristics (ROC) curves where sensitivity is plotted against the error rate (percentage of false positive). ROC_*n *_statistics for individual methods are also provided in Table 1. As can be seen from Figure 1 and Table 1, SPEER, GroupSim and MultiRELIEF clearly perform better than the other two methods with their sensitivities at 5% error rate being 54, 38 and 40 respectively (Additional file 1). Similar trend is also observed in PR (precision-recall) curves where precision (TP/TP+FP) for each method is plotted on the *y*-axis, and recall (TP/TP+FN) is plotted on the *x*-axis (Additional file 2). It should be mentioned that the SPEL method does not take full advantage of the curated subfamily clustering provided in the validation testset since SPEL performs the clustering automatically along with the subsite identification. If there is no information on subfamilies, the automatic clustering is advantageous, but this is not within the scope of our paper to analyze such cases.

**Table 1:** Comparison of ROC_*n *_statistics for different methods (see Methods for definition).

**Methods**	**ROC**_100_	**ROC**_200_	**ROC**_500_	**ROC**_1000_	**ROC**_3000_
SPEER	0.12 ± 0.009	0.22 ± 0.014	0.40 ± 0.011	0.54 ± 0.009	0.80 ± 0.005
GroupSim	0.08 ± 0.011	0.20 ± 0.013	0.36 ± 0.011	0.53 ± 0.009	0.78 ± 0.005
MultiRELIEF	0.11 ± 0.010	0.16 ± 0.009	0.32 ± 0.014	0.50 ± 0.011	0.78 ± 0.006
SDPpred	0.04 ± 0.005	0.08 ± 0.008	0.22 ± 0.008	0.33 ± 0.005	0.70 ± 0.007
SPEL*	0.02 ± 0.010	0.06 ± 0.008	0.16 ± 0.010	0.30 ± 0.012	0.62 ± 0.005

* SPEL algorithm performs automatic clustering along with the subsite identification.

### Prediction of potential subsites and their structural properties

Based on the performance assessment using the validation dataset (195 subsites from 20 family alignments) three best performing methods, namely SPEER [20] GroupSim [16] and MultiRELIEF [17] were further employed to identify new potential subsites. Results (top 15 predicted sites excluding the actual subsites) from these three methods were compared and sites that were commonly predicted by all three methods (C3 sites) or by any pair of methods (C2 sites) were selected as new potential subsites. Additional file 3 provides a list of such 264 new potential subsites (135 C3 sites, 129 C2 sites) for all families.

Since the sets of C3 and C2 sites do not include actual subsites and are not assigned any combined rank and score (this would require combining scores from different methods which is a non-trivial task), it is difficult to validate the performance of the ensemble approach. To estimate the performance, we defined subsites predicted by three or two methods (top 15 predicted sites including actual subsites; C3 and C2 sites). Altogether we identified 141 such C3 and 129 C2 sites, calculated the PR statistics and compared it with each individual method (Additional file 4). Expectedly, C3' and C2' sites provide better reliability (precision) than sensitivity (recall) compared to individual prediction methods.

### Distribution of spatial distances

Understandably, experimental validation is the most authentic verification process for the predicted subsites. But, in the absence of such rigorous protocol one alternate way would be to examine structural features which are characteristic for actual subsites (such as the distribution of their spatial distances, solvent accessibility, secondary structural content and hydrogen bonding patterns) and to compare them with the characteristic structural features of predicted subsites.

**Figure 2:**
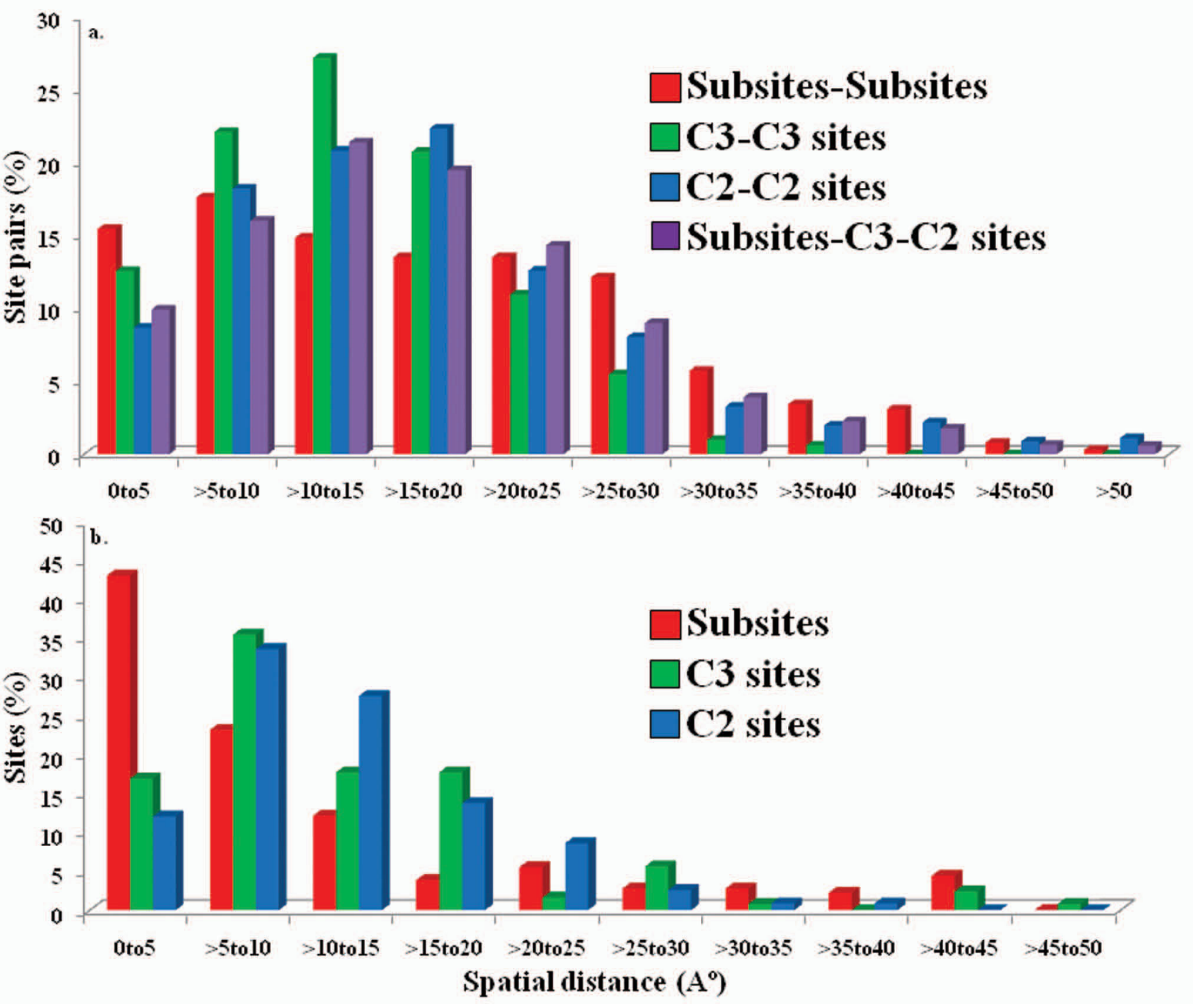
**Distribution of spatial distances between actual and potential subsites**. (a) and distances of actual/potential subsites to the specific ligand/substrate (b) for validation test set. Distances were calculated using the nearest protein and ligand/cofactor atom coordinates supplied in the individual PDB files. Potential subsites, commonly predicted either by all three best methods are called C3 sites or by any two methods are called C2 sites.

Figure 2 shows the distribution of spatial distances between actual and between potential subsites (Figure 2a); distances of actual/potential subsites to the specific ligand/substrate (Figure 2b). As can be seen from Figure 2a, the mode of the pairwise distance distribution of the actual subsites is shifted toward lower distances compared to C3-C3 distances and this shift is more pronounced with respect to C2-C2 distances. Indeed, majority of site pairs fall within 20 Å and within this distance range the distribution means are statistically different (p-value << 10^-5^). Interestingly enough, for distances less than 20 Å, the more reliable prediction method is used (C3 instead of C2 sites), the closer potential subsites are to each other and to the distance distribution of actual subsites. For distances larger than 20 Å the situation is different and the actual subsite distance distribution has a longer tail corresponding to subsites located at large distances from each other.

Figure 2b shows the spatial distances of actual and potential subsites from the specific substrate/ligands. As can be seen from this figure, the larger fraction (66%) of actual subsites is found to be in close contact (<= 10 Å) to substrates/ligands compared to C3 and C2 sites (52 and 46% respectively). This difference is even more prominent at a closer range (<= 5 Å) where 43% of actual subsites are found compared to only 17% C3 and 12% C2 sites. This might indicate the possibility of indirect interactions of C3 and C2 sites with the specific substrate/ligands. It shows that combining more reliable methods' predictions (C3 sites) provides better agreement with the actual subsite-ligand distance – another indication that the analysis of distance distribution patterns can provide the means to validate the prediction accuracy.

### Structural properties of actual and predicted subsites

**Figure 3:**
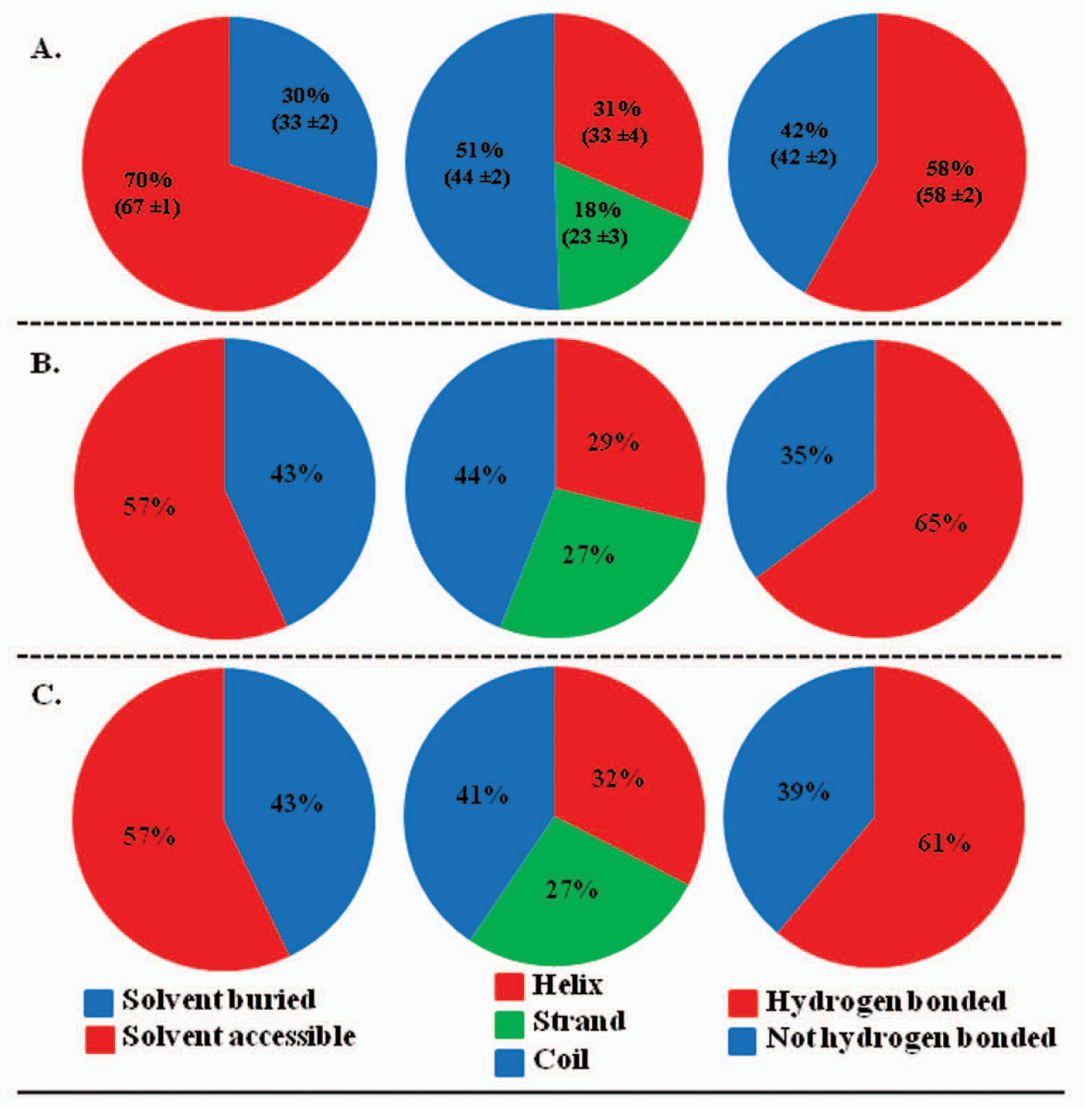
**Structural properties of actual and potential subsites (C3 and C2 sites)**. Solvent accessibility, secondary structure content and hydrogen bonding patterns for actual subsites (A), C3 (B) and C2 sites (C) were computed from the individual protein structures using the JOY package [28]. Structural property values extracted from all residues in our dataset are mentioned within parenthesis.

Important structural characteristics such as solvent accessibility, secondary structural content and hydrogen bonding patterns of actual and predicted subsites were analyzed and compared. Figure 3 shows the solvent accessibility, secondary structure content and hydrogen bonding patterns of actual subsites (a), C3 (b) and C2 (c) sites. Overall, the distributions of structural properties of potential subsites are not very different from that observed for actual subsites or all sites. As can be seen from this figure, subsite prediction methods tend to over predict sites in beta-strands and under predict sites in solvent accessible areas and coils which are less evolutionary conserved than protein cores.

### Examples of predicted subsites

**Figure 4:**
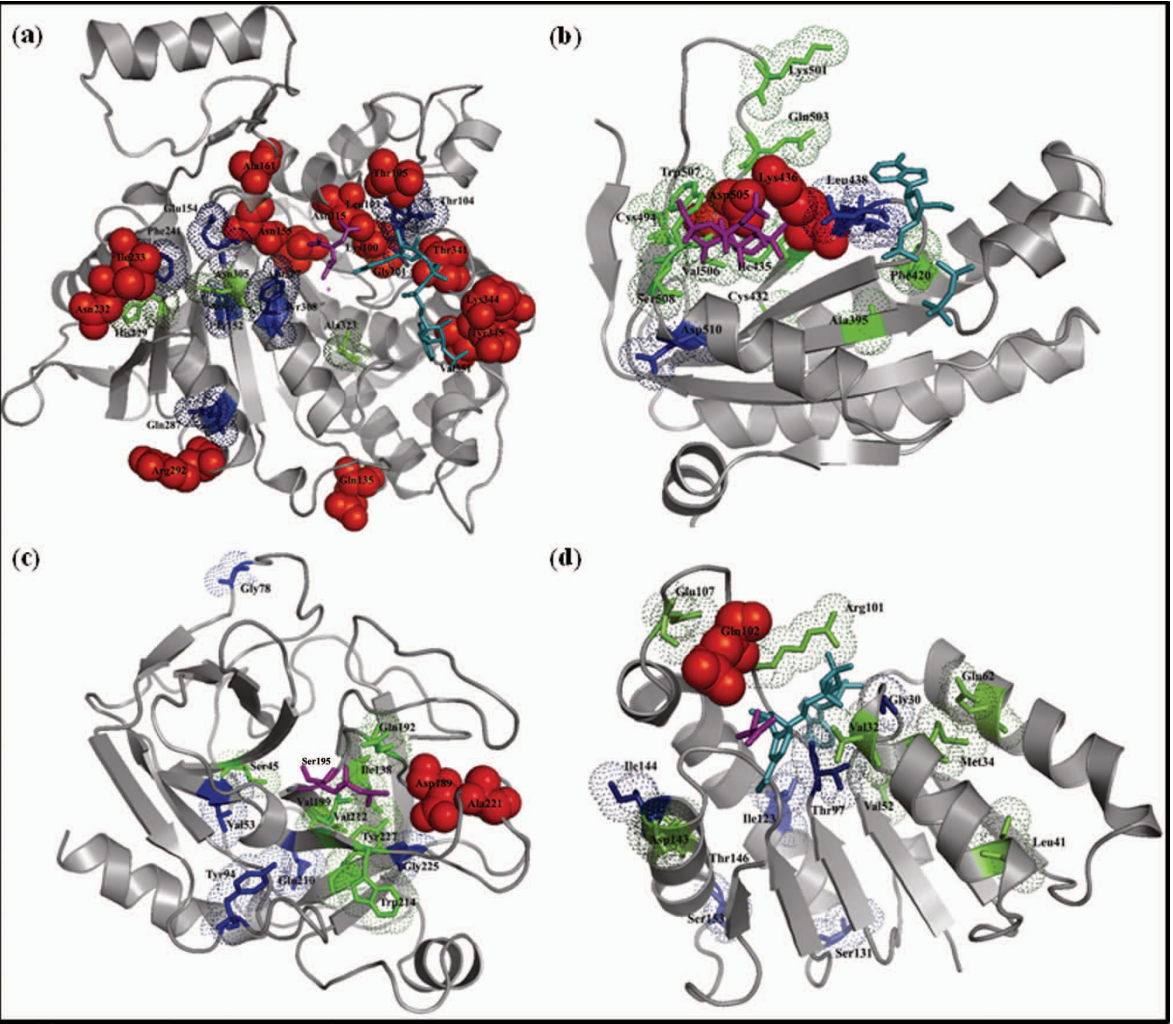
**Actual and potential subsites are mapped onto the representative 3D structures from four families in the validation dataset**. Actual subsites are shown in space fill model and colored in red while subsites that were commonly predicted by all three methods (C3 sites) or by any pair of methods (C2 sites) are shown in stick and colored in green and blue, respectively. Specific ligands and/or cofactors are shown in purple and cyan. a) IDH_IMDH family (1AI2; ligand: isocitrate; cofactor: NADP) b) nucleotidyl cyclase family (1CS4, chain A; activator: forskolin; P-site inhibitor: 2'-d-3'-AMP.PPi) c) serine protease family (5PTP; ligand: specificity determining serine residue at position 195) and d) LDH_MDH family (9LDT, chain A; ligand: oxamate; cofactor: NAD). The figure was generated using the PyMOL software [23].

Actual and potential subsites are shown for four protein families in Figure 4. For the IDH_IMDH family, SPEER, GroupSim and MultiRELIEF identified 10, 8 and 6 actual subsites, respectively, at 15% error rate. However, three other sites (N305, H229, and A323) were commonly predicted by all three methods (within the top 15 predicted sites excluding actual subsites). Figure 4a maps the actual subsites along with sites that were commonly predicted by all three (three C3 sites; colored in green) or any two methods (nine C2 sites; colored in blue) onto 3D-structure of a representative protein from IDH_IMDH family. Spatial mapping of the potential subsites shows that two (N305 and A323) of the three C3 sites, reside within close distance (<= 10 Å) with respect to the specific cofactor NADP (shown in cyan) or specific ligand, isocitrate (shown in purple). In addition, five C2 sites (G101, L103, T104, E154, and Y308) are also found to be less than 10 Å apart in space from the NADP or isocitrate molecule.

For nucleotidyl cyclase family both actual subsites were identified by SPEER, GroupSim and MultiRELIEF within 15% error rate (Figure 4b). Eight potential C3 sites and three C2 sites fall within 10 Å distance from the specific activator (forskolin; shown in purple) or P-site inhibitor molecules (2'-deoxy-3'-AMP and pyrophosphate; shown in cyan).

SPEER and GroupSim successfully predicted both actual subsites (D189 and A221) for the serine protease family while MultiRELIEF failed to identify one subsite (D189) within 15% error rate. However, there are seven sites besides actual subsites that were commonly predicted by all three methods. Figure 4c provides a representative structure of trypsin with the actual subsites and commonly predicted subsites (C3 and C2 sites). All C3 sites reside less than 10 Å apart from the specificity determining serine residue (marked in purple) whereas three C2 sites reside within 5 Å from the serine residue.

Finally, nine C3 and seven C2 sites were identified for the lactate-malate dehydrogenase (LDH_MDH) family. Figure 4d shows a representative structure of lactate dehydrogenase complexed with cofactor, NAD (marked in cyan in Figure 4d) and ligand, oxamate. Predicted C3 and C2 sites were also projected onto the lactate dehydrogenase structure. 3D structural images were generated using the PyMOL software [23].

### Prediction of potential subsites using automatic family clustering

**Figure 5:**
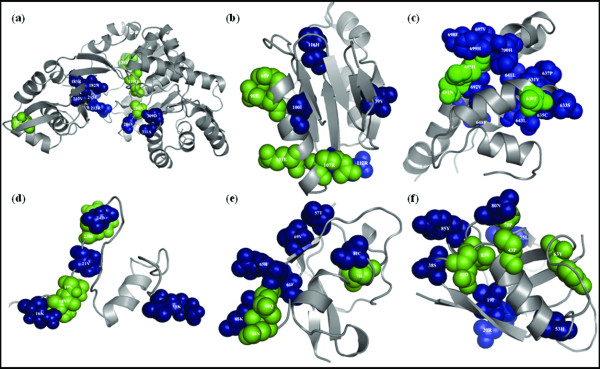
**Projection of potential subsites onto the representative structures of families in prediction testset**. Potential subsites commonly identified by three methods, SPEER, GroupSim, and MultiRELIEF are mapped onto representative structures from the following families: a) ADP specific phosphofructokinase/glucokinase family (1U2X); b) DUF498/DUF598 domain family (1IHN); c) guanine nucleotide exchange factor (GEF)-Ras like GTPases family (1NVX); d) p21-Rho binding domain family (1CEE); e) Raf-like Ras-binding domain family (1C1Y) and f) Ras association (RalGDS/AF-6) domain (1LFD) family. Commonly predicted C3 and C2 sites are colored in green and blue, respectively. The figure was generated using the PyMOL software [23].

To check whether the use of automatic family clustering and the lack of manual curation would affect the subsite prediction accuracy, we predicted subsites for six protein families obtained from Proteinkeys database (Additional file 5; prediction dataset) that have automatically defined subgroups with at least three protein sequences. Three best performing prediction methods (SPEER, GroupSim and MultiRELIEF) were applied to this testset to identify potential new candidate subsites for specificity determination (Additional file 6). Since there is no information on the actual subsite locations for the automatically determined alignments from "prediction testset", we applied structural analysis of C3 and C2 sites which, as was shown in the previous section, may be indirectly used to validate the subsite prediction accuracy. Potential subsites for the six families as suggested by common prediction of all three methods or any two methods are listed in Additional file 6. In total, 24 C3 and 47 C2 sites were identified for the six families. These identified C3 and C2 sites could be extremely important in determining the specificity and therefore can be ideal target for mutagenesis experiments. Figure 5 provides projection of these predicted C3 and C2 sites onto representative structures from six families. Commonly predicted C3 and C2 sites are shown in space filling model and are colored in green and blue, respectively. 3D structural images were generated using the PyMOL software [23].

Spatial distances among the C3 and C2 sites were also analyzed. It has been observed that 90% of C3 sites are located within 20 Å distance with respect to each other (Additional file 7) whereas 80% of C2 sites reside within 20 Å distance. Overall, we observed similar distributions of structural properties of potential (C3 and C2) subsites from prediction testset and C3 and C2 sites identified from validation testset (Figure 2, Additional file 8). One exception is the solvent accessibility which tends to be larger for potential sites from the prediction testset.

## Discussion

It is extremely difficult task to detect features that are responsible for protein functional divergence and further differentiate evolutionary changes leading to new specificities. Indeed, despite numerous efforts in predicting the specificity determining sites, the accuracy remains limited as most methods are not sensitive enough to detect small variations between subsites. An alternate approach to the underlying problem has been examined here, where several prediction methods were simultaneously employed for detection of subsites within protein families. This ensemble approach combining best performing methods not only validates the performance of the available prediction methods but, also provides reliability of the new prediction. The performance of five methods, namely, SPEER [19] GroupSim [15], MultiRELIEF [16], SDPpred [11], and SPEL [17] were evaluated in 20 well studied protein families. These families provide accurate alignments, reliable subgroup identifications and the locations of subsites. Although all methods identified majority of actual subsites, SPEER, GroupSim and MultiRELIEF performed better and reached reasonable sensitivities of 54%, 38% and 40% at 5% error rates. Importantly, several other sites (Additional file 3) were commonly predicted by three best performing methods (135 C3 sites) or by any pair of methods (129 C2 sites). These sites might also be important in determining the specificity and therefore can be ideal targets for mutagenesis experiments.

## Conclusion

Our analysis of structural characteristics first showed that if we use an ensemble of three best methods, the distance distribution of potential subsites has a higher similarity to the distribution of actual subsites for distances less than 20 Å. Interestingly, we observed a smaller fraction of C3 and C2 sites at larger distances compared to the actual subsites. We observed a similar distribution pattern while studying the coevolution of subsites in our previous work [24] with 40% of coevolved subsite pairs located at distances more than 20 Å. Possible explanations for this long-range evolutionary coupling can include the possibility of allosteric regulation, or formation of nonsymmetrical homodimers. As judged from the comparison of distance distributions, the subsites which are closer to each other in space seem to be easier to predict by existing methods. The analysis of hydrogen bonding, solvent accessibility and secondary structure content showed that, overall, distributions of structural properties are quite similar for actual and potential subsites indicating their similar involvement in determining the specificity in protein families.

Further, new potential subsites were predicted for six other protein families where subgrouping of the sequences was done by an iterative automated clustering optimization procedure. Even though the actual subsites were not available for these families, we were able to observe certain similarities of structural features between predicted and actual subsites which might point to their functional relevance.

## Methods

### Datasets of protein families and subsites

Reliable manually curated alignments were collected from different sources for 20 families for which experimental evidence was available on the locations of most of the subsites (see Additional files 9 and 10 for description). Each of these families (along with their subsites) was previously used in separate studies [20,11-13,17,18,25]. The alignments were constructed by existing alignment methods and were subjected to the additional round of careful manual curation. The family alignments were grouped into subfamilies based on different criteria including sequence and structural properties, kinetic properties, substrate specificity, taxonomy, and function [20,11-13,17,18,25].

In this study the experimentally annotated subsites ("actual" subsites, 195 in total) were pooled together and used as gold standards to compare and validate the performance of several prediction methods ("validation testset"). Details about the families can be found in Additional file 11. Alignments, location of subsites and the SPEER program are available via .

We also predicted subsites for six protein families obtained from Proteinkeys database, version 0.81 beta  where sequence subgrouping was done by the automated clustering procedure ("prediction testset"). Subgrouping of the sequences in Proteinkeys database was done by an iterative automated optimization procedure to cluster similar sequences with optimal separation. Each individual family alignment was clustered into subgroups through utilization of optimization coefficient A (0<A<1). Typically, the optimal value of A falls between 0.65 and 0.85, therefore a default value of A was taken to be 0.75. Six families from "prediction testset" which were used to identify new potential subsites included: ADP specific phosphofructokinase/glucokinase, DUF498/DUF598, guanine nucleotide exchange factor(GEF)-Ras like GTPases, p21-Rho binding domain, Raf-like Ras-binding domain, and Ras association (RalGDS/AF-6) domain families. Only those Proteinkeys families were selected for the current study where total number of subgroups did not exceed twenty and every subgroup was represented by at least three protein sequences (altogether six families in prediction dataset; Additional file 5).

### Subsite prediction methods

Five computational methods, SPEER [20] GroupSim [16], MultiRELIEF [17], SDPpred [12] and SPEL [18] were applied on the validation dataset (20 families; 195 subsites) to identify the subsites. We also tried to employ two other methods, Sequence Harmony (SH; 13) and Treedet [9,10]. However, SH works only for families with two subgroups whereas 30% of our dataset families contain more than 2 subgroups or subfamilies. Similarly, Treedet server is restricted to input alignment length and provided (using default parameters) results for only 50% of the families within the validation dataset. Therefore, prediction results from these two methods were not included in this study.

Any individual method does not perform equally well to identify all different types of specificity determining sites [20] and those sites which are predicted simultaneously by several top methods are more reliably predicted compared to only one method used. Given that the prediction methods perform better than random any combined approach should be an improvement. **S**ubsequently, results from the three best performing methods in the validation test, namely SPEER, GroupSim, and MultiRELIEF were compared and combined to predict potential subsites in addition to the actual subsites. Similarly, these three methods were also applied on the prediction testset obtained from the Proteinkeys database. A short description of each method is given below. The perl script providing such combined approach in predicting subsites is available at . To distinguish subsites from globally conserved sites we excluded from the subsite set those highly conserved positions within the overall alignment where any amino acid type was represented more than 80% of the time (only one highly conserved subsite was present among 195 actual subsites).

### SPEER (Specificity prediction using amino acids' Properties, Entropy and Evolution Rate)

SPEER [20] combines Euclidean distances based on amino acids' physico-chemical properties, evolutionary rate and combined relative entropy to predict subsites. All three terms account for the variability of sites within the subfamilies in terms of their physico-chemical properties, evolutionary rates and amino acid types. The first and the third terms also approximate the variability of physico-chemical properties and amino acid types between the subfamilies. As the background conservation levels may vary substantially between different protein families, each of the three scores is normalized using the background score distribution of the family alignment. The linear combination of three normalized scores, termed as SPEER score is used to predict the subsites.

### GroupSim

GroupSim [16] is a sequence based subsite prediction method, which compares average similarity of amino acids within and between subgroups. The average similarity of amino acids is calculated using a similarity matrix (identity matrix) for each subgroup in the alignment. The GroupSim score is the average within-group similarity minus the average between-group similarity. Higher scores indicate a greater likelihood to be a subsite. This program also employs an accessory heuristic module, 'ConsWin' that considers sequence conservation of neighboring amino acids as well.

### MultiRELIEF

MultiRELIEF [17] uses 'local' sequence conservation properties for identification of subsites. This approach utilizes a machine learning technique for feature weighting, called RELIEF, which exploits the 'local' sequence space for discriminating samples (sequences) from two subgroups [26,27]. RELIEF assigns a weight to features (sites) according to their ability to separate different samples or subgroups. The subsites are predicted based on the maximum weight which is calculated iteratively as a Hamming distance between a given sequence and the nearest sequence from another subgroup minus Hamming distance between a given sequence and its nearest neighbor from the same subgroup. MultiRELIEF can handle multiple subgroups by random sub-sampling of pairs of subgroups. It should be mentioned that MultiRELIEF can also exploit 3D structure information by increasing the weight of residues that have high number of contacts with other residues. However, this option is not used in the current study as none of the other methods use additional structural information, which is not always available.

### SDPpred (Specificity Determining Position prediction)

SDPpred [12] utilizes mutual information to identify the positions that are conserved within the subgroups but differ between them. SDPpred takes into account the similarity between the amino acids in the form of amino acid substitution matrices. To estimate the statistical significance of the obtained values of mutual information, it shuffles each column to calculate the Z-score. SDPpred also attempts to account for the background similarity between proteins by calculating the expected mutual information for each column.

### SPEL (Specificity Positions by Evolutionary Likelihood)

SPEL [18] utilizes evolutionary log-likelihood of amino acid distribution to detect subsites. It should be mentioned that SPEL does not require a predetermined subgroup definition which puts it in the separate group compared to other tested methods. A phylogenetic tree is reconstructed from the multiple sequence alignment, and P-values of an evolutionary likelihood-based score for alignment positions are estimated from a random model that eliminates any functional specificity signal. Positions with low P-values are likely to be important for functional specificity.

### Evaluation of prediction accuracy

The performance of various prediction methods were evaluated using the actual subsite information from validation dataset and by calculating the Receiver Operating Characteristics (ROC) curves and ROC statistics. For a given alignment, the sensitivity and error rates were estimated based on the number of true positives (correctly predicted actual subsites) and false positives (incorrectly predicted actual subsites) found above each score cutoff. Sensitivity (TP/TP+FN) was defined as the number of true positives (TP) found at each score threshold divided by the sum of true positives and false negatives (FN), where false negatives are defined as actual subsites below the score threshold. Error rate (FP/FP+TN) was estimated as the number of false positives (FP) divided by the sum of false positives and true negatives (TN, non-subsites below the score threshold). Each method's performance was also evaluated by estimating the ROC_*n *_statistics [28] where the sum of the number of true positives corresponding to 1, 2, 3,...*n *false positives on the ROC curve (*t*_i_) was normalized by the sum of true positives and false negatives: *T *= *(TP+FN)*, *ROC*_*n *_= (Σ_i = 1,...,*n *_*t*_*i*_*)/nT*. Standard deviations of ROC statistics were estimated analytically using expressions provided in Schaffer *et al*, 2001 [28]. Precision (TP/TP+FP) and Recall or Sensitivity (TP/TP+FN) curve was also derived to compare the performance of each method.

### Calculation of spatial distances and structural properties

Representative 3D structures were collected for each family from the PDB database [29]. Spatial distances were calculated using atom coordinates supplied in the individual PDB file. Structural properties such as solvent accessibility, secondary structures, and hydrogen bonds were computed from the protein structure using the JOY package [30]. Solvent accessibility was measured using the PSA program from the JOY package and residues that have an accessible surface area less than 7% were treated as solvent buried or inaccessible. Similarly, secondary structures (helix, strand and coil) and hydrogen bonding patterns were estimated using the SSTRUC and HBOND programs from the JOY package [30], respectively.

## Authors' contributions

SC and AP conceived of the study and wrote the manuscript. SC carried out the methods' comparisons and subsite validation. All authors have read and approved the final manuscript.

## Supplementary Material

Additional file 1 **Ensemble approach to predict specificity determinants: benchmarking and validation**. Table listing prediction sensitivities of different methods. Click here for file

Additional file 2 **Ensemble approach to predict specificity determinants: benchmarking and validation**. Precision-Recall (PR) curves. Click here for file

Additional file 3 **Ensemble approach to predict specificity determinants: benchmarking and validation**. List of potential subsites identified in validation dataset. Click here for file

Additional file 4 **Ensemble approach to predict specificity determinants: benchmarking and validation**. Comparison of performance of different methods. Click here for file

Additional file 5 **Ensemble approach to predict specificity determinants: benchmarking and validation**. Prediction dataset. Click here for file

Additional file 6 **Ensemble approach to predict specificity determinants: benchmarking and validation**. List of potential subsites predicted in prediction dataset. Click here for file

Additional file 7 **Ensemble approach to predict specificity determinants: benchmarking and validation**. Spatial distances among the predicted C3 and C2 sites for six families within the prediction dataset. Click here for file

Additional file 8 **Ensemble approach to predict specificity determinants: benchmarking and validation**. Structural property analysis of potential (C3 and C2 sites) subsites within the prediction dataset. Click here for file

Additional file 9 **Ensemble approach to predict specificity determinants: benchmarking and validation**. Validation dataset. Click here for file

Additional file 10 **Ensemble approach to predict specificity determinants: benchmarking and validation**. List of actual subsites. Click here for file

Additional file 11 **Ensemble approach to predict specificity determinants: benchmarking and validation**. Additional information about validation dataset. Click here for file
