# Supplementary material for: Ensemble approach to predict specificity determinants: benchmarking and validation
Source: BMC Bioinformatics. 2009 Jul 2;10:207. doi: 10.1186/1471-2105-10-207 (PMC2716344; doi:10.1186/1471-2105-10-207)
Supplement: Additional file 1 — Ensemble approach to predict specificity determinants: benchmarking and validation. Table listing prediction sensitivities of different methods. [file 1471-2105-10-207-S1.doc]

**Additional file 1: Prediction sensitivities**

| **Error rate** | **Prediction sensitivity (%)** | | | | |
| --- | --- | --- | --- | --- | --- |
|  | **SPEER** | **GroupSim** | **MultiRELIEF** | **SDPpred** | **SPEL** |
| **1%** | 17 | 13 | 11 | 5 | 7 |
| **5%** | 54 | 38 | 40 | 36 | 30 |
| **15%** | 70 | 70 | 60 | 58 | 50 |
