# Supplementary material for: Ensemble approach to predict specificity determinants: benchmarking and validation
Source: BMC Bioinformatics. 2009 Jul 2;10:207. doi: 10.1186/1471-2105-10-207 (PMC2716344; doi:10.1186/1471-2105-10-207)
Supplement: Additional file 2 — Ensemble approach to predict specificity determinants: benchmarking and validation. Precision-Recall (PR) curves. [file 1471-2105-10-207-S2.doc]

**Additional file 2**

**
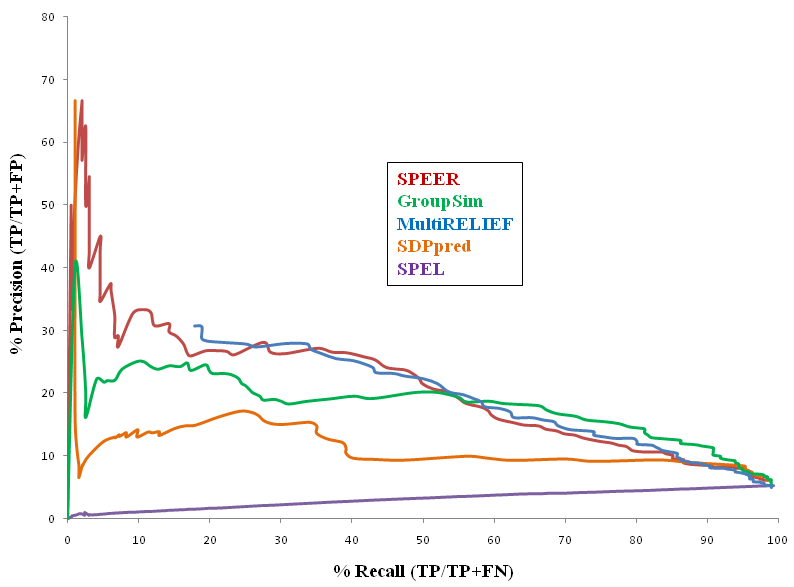
**

Comparison of prediction performances. Precision-Recall (PR) curves for prediction of subsites are shown for SPEER, GroupSim, MultiRELIEF, SDPpred and SPEL methods.
