# Supplementary material for: Ensemble approach to predict specificity determinants: benchmarking and validation
Source: BMC Bioinformatics. 2009 Jul 2;10:207. doi: 10.1186/1471-2105-10-207 (PMC2716344; doi:10.1186/1471-2105-10-207)
Supplement: Additional file 3 — Ensemble approach to predict specificity determinants: benchmarking and validation. List of potential subsites identified in validation dataset. [file 1471-2105-10-207-S3.doc]

**Additional file 3**: List of potential subsites identified in validation dataset.

| **Family code** | **Common in all three methods***  **(C3 sites)** | **Common in any two methods***  **(C2 sites)** | **Ranks of C3 sites^#^**  **SPEER|GroupSim|MultiRELIEF** | **Ranks of C2 sites^#^**  **SPEER|GroupSim|MultiRELIEF** |
| --- | --- | --- | --- | --- |
| CBM9 | 174S|0|C|Hy|5.51; 103N|1|C|Hy|15.34; 120V|0|E|Hn|17.19; 73Q|1|C|Hy|9.22; 173N|1|C|Hy|6.10; 75S|0|E|Hn|8.41; 119A|1|E|Hy|18.66; 69N|1|C|Hy|7.24 | 72E|1|C|Hy|6.76; 118T|0|E|Hy|14.83; 177D|1|C|Hy|8.89; 123I|1|E|Hn|22.11; 56A|0|E|Hn|14.92; 12D|1|C|Hy|23.79 | 1|2|8; 2|12|14; 3|3|2; 4|5|11; 5|4|7; 6|11|4; 8|1|6; 9|8|10 | 7|6|NF; 12|10|NF; 15|7|NF; 11|NF|15; 14|NF|9; NF|12|15 |
| GST | 13T|0|C|Hy|6.46; 10R|0|C|Hy|5.85; 2M|1|E|Hn|18.16 | 71Q|1|C|Hy|8.62; 87G|0|C|Hn|24.38; 97D|1|H|Hy|17.79; 80A|0|H|Hy|19.62; 64D|1|E|Hy|17.73; 157F|0|H|Hy|13.17; 61Y|0|E|Hn|8.13; 14H|0|H|Hn|8.85; 148A|0|C|Hn|22.91 | 1|7|6; 3|15|13; 4|6|2 | 11|4|NF; 13|9|NF; 14|2|NF; 15|14|NF; 2|NF|3; 5|NF|1; 6|NF|5; 12|NF|12; NF|10|10 |
| Gprotein | 30V|1|C|Hn|13.75 | 147S|1|C|Hn|13.77; 176K|1|C|Hn|2.93; 307N|0|C|Hy|12.40; 267K|1|H|Hy|22.44; 321C|1|C|Hy|19.51; 270F|0|H|Hn|20.98; 143Q|1|C|Hy|5.68; 192F|0|E|Hn|14.71; 139A|1|C|Hy|10.57; 205K|1|C|Hn|2.92 | 8|8|10 | 2|1|NF; 5|2|NF; 7|12|NF; 14|7|NF; 1|NF|4; 4|NF|7; 9|NF|8; 12|NF|9; 13|NF|14; NF|13|5 |
| LacI | 20H|1|H|Hy|5.32 | 302F|0|H|Hy|17.92; 69S|1|C|Hn|8.35; 36V|0|H|Hy|7.88; 21V|0|H|Hy|7.38; 96N|1|E|Hn|10.93; 246G|1|C|Hy|5.45; 66A|0|E|Hy|9.77; 11A|0|H|Hy|7.08; 91T|1|E|Hy|18.57 | 1|1|6 | 2|14|NF; 3|4|NF; 5|3|NF; 6|15|NF; 7|2|NF; 9|5|NF; 12|6|NF; 4|NF|3; 11|NF|7 |
| CNmyc | 413, 259, 494 497, 404, 507, 491, 505, 410, 254, 566 | 21, 511, 53, 569, 162 |  |  |
| cd00120 | 18Q|1|H|Hy|--; (37V)|1|H|Hy|-- | 4K|1|C|Hn|--; 44A|1|E|Hn|--; 53K|1|C|Hn|--; 19V|1|H|Hy|--; 54L|1|E|Hn|--; 57Y|1|E|Hn|--; 50S|1|C|Hy|--; 52N|1|C|Hy|--; 39C|1|C|Hn|--; 37V|1|H|Hy|--; 41C|1|C|Hy|-- | 2|1|1; 6|4|13 | 1|2|NF; 5|6|NF; 3|NF|5; 4|NF|9; 7|NF|4; 8|NF|11; 12|NF|3; 13|NF|12; 15|NF|2; NF|14|3; NF|6|9 |
| cd00264 | 66S|0|E|Hn|7.41; 189Y|0|H|Hy|3.63; 200D|1|C|Hy|4.19; 209L|0|C|Hn|6.61; 4G|0|C|Hy|11.80 | 144V|0|E|Hy|28.76; 105D|1|E|Hy|26.15; 35P|1|C|Hn|21.47; 191Q|1|C|Hy|8.03; 217A|1|C|Hn|13.82; 135C|0|E|Hn|9.59; 192T|1|C|Hy|8.49 | 1|2|6; 3|3|14; 4|1|3; 5|5|8; 6|15|4 | 2|11|NF; 7|NF|7; 9|12|NF; 10|4|NF; 12|14|NF; 13|13|NF; NF|15|6 |
| cd00333 | 186L|1|H|Hy|8.39; 171N|1|C|Hy|18.44 | 192A|1|H|Hy|9.18; 232Y|0|C|Hn|6.78; 142H|1|C|Hy|6.73; 17G|0|H|Hy|7.08; 166L|1|H|Hy|10.75; 190I|0|H|Hy|7.95; 233F|1|C|Hy|12.26; 197L|1|H|Hy|4.03; 85K|1|C|Hy|14.20; 45S|0|H|Hy|6.58 | 1|2|6; 3|12|2 | 2|4|NF; 4|11|NF; 5|14|NF; 6|1|NF; 7|13|NF; 10|7|NF; 8|NF|4; 13|NF|5; 14|NF|9; NF|13|15 |
| cd00363 | 137I|0|C|Hy|7.71; 251Q|0|C|Hy|6.82; 156T|1|H|Hy|9.05; 136T|0|C|Hy|7.75; 246V|1|E|Hn|10.38; 151D|1|H|Hy|8.88 | 16M|0|H|Hy|6.49; 17N|0|H|Hy|13.52; 66T|0|C|Hy|10.37; 264A|0|H|Hy|14.73; 75E|1|C|Hn|5.44; 18A|0|H|Hy|15.52; 94I|0|C|Hn|17.49; 8T|0|E|Hn|7.88; 124G|0|C|Hn|4.92; 19A|0|H|Hy|13.52; 247L|0|C|Hn|9.85; 71A|1|C|Hn|4.52 | 3|6|7; 4|5|2; 6|13|5; 7|9|8; 10|10|4; 11|12|6 | 1|2|NF; 2|3|NF; 12|4|NF; 15|7|NF; 5|NF|14; 8|NF|1; 9|NF|12; 13|NF|10; NF|9|1; NF|15|8; NF|3|11; NF|13|14 |
| cd00365 | 657M|1|H|Hy|26.77; 766Q|0|C|Hy|18.92; 686N|1|C|Hn|26.61; 769A|1|C|Hy|16.77; 751A|0|C|Hy|3.35; 862L|1|H|Hy|3.61; 850E|0|H|Hy|7.24 | 558T|1|C|Hn|8.60; 775S|1|C|Hy|11.33; 772V|1|H|Hn|14.17; 748G|1|E|Hy|8.86; 739G|1|H|Hy|9.67 | 1|1|12; 2|2|3; 3|8|11; 4|4|2; 5|3|4; 6|10|1; 10|7|13 | 7|6|NF; 9|9|NF; 11|5|NF; 15|15|NF; 8|NF|15 |
| cd00423 | 140H|0|C|Hn|10.76; 94S|0|E|Hn|14.56; 47M|0|H|Hy|12.61; 26D|1|C|Hy|13.20; 97T|0|C|Hy|13.65; 77R|0|H|Hy|17.47 | 116D|0|E|Hy|12.62; 20I|0|E|Hy|6.75; 58G|0|E|Hn|11.85; 189G|1|C|Hn|5.73; 258D|1|C|Hn|8.33; 25P|1|C|Hn|14.84; 187G|0|C|Hn|8.75 | 2|9|6; 3|5|1; 5|14|10; 6|4|2; 7|6|14; 10|11|3 | 1|1|NF; 4|2|NF; 9|3|NF; 11|10|NF; 12|12|NF; NF|5|7; NF|4|8 |
| cd00985 | 136G|1|E|Hy|12.77; 181A|0|H|Hy|9.58; 88Y|1|C|Hy|6.91; 177H|0|H|Hy|2.54; 178R|0|H|Hy|2.31; 23E|1|H|Hy|3.03; 172K|1|H|Hy|7.38; 89S|0|H|Hy|2.75; 86G|1|E|Hy|5.91; 72E|0|E|Hy|4.88; 85P|0|E|Hn|7.30; 149F|1|C|Hn|3.39; 113A|0|E|Hy|10.82 | 75G|0|E|Hy|3.15 | 1|4|2; 2|1|1; 3|2|4; 4|5|9; 5|3|8; 6|7|7; 7|9|3; 8|8|6; 9|6|10; 10|10|15; 11|11|14; 12|12|5; 14|15|11 | 15|14|NF |
| Ricin | 388Q|0|C|Hy|19.50; 401V|1|C|Hn|17.22; 337V|0|C|Hy|8.52; 392L|1|E|Hn|17.65; 316K|1|E|Hn|13.19; 357E|0|C|Hy|19.35; 387N|1|C|Hy|17.99 | 409A|0|E|Hy|3.28; 351T|1|E|Hn|15.31 | 1|13|1; 2|1|4; 3|6|3; 4|2|5; 6|4|8; 8|9|11; 10|3|15 | 12|5|NF; 9|NF|2 |
| IDH_IMDH | 305N|1|H|Hy|8.99; 229H|0|E|Hy|17.12; 323A|1|C|Hn|7.42 | 154E|0|E|Hy|8.34; 38G|0|H|Hn|7.83; 287Q|1|H|Hy|17.31; 101G|0|C|Hn|5.13; 241F|0|H|Hy|15.40; 152F|0|E|Hn|11.97; 103L|0|C|Hn|3.39; 308Y|1|H|Hn|6.31; 104T|1|C|Hn|3.14 | 1|1|11; 4|8|15; 8|5|10 | 10|11|NF; 14|4|NF; 15|7|NF; 3|NF|1; 5|NF|2; 11|NF|6; 12|NF|8; NF|12|2; NF|7|6 |
| LDH_MDH | 143D|1|H|Hy|7.12; 107E|1|C|Hy|8.25; 62E|1|H|Hy|8.09; 101R|1|C|Hy|2.82; 32V|1|H|Hy|2.83; 34M|1|H|Hn|6.33; 148V|1|H|Hn|12.33; 41L|0|H|Hy|15.92; 52V|0|E|Hy|4.19 | 123I|0|H|Hy|3.49; 30G|0|C|Hy|3.49; 153S|0|H|Hn|14.84; 131S|0|C|Hy|14.94; 144I|1|H|Hy|9.62; 146T|1|H|Hy|8.17; 97T|0|C|Hn|2.93 | 1|1|1; 2|5|7; 3|3|10; 4|4|2; 5|2|11; 6|8|4; 7|7|5; 10|11|3; 13|12|9 | 9|6|NF; 14|14|NF; 8|NF|6; 11|NF|12; 12|NF|15; NF|14|10; NF|8|13 |
| Nucleotidyl_Cyclase | 506V|0|E|Hn|2.79; 420F|1|H|Hy|9.23; 507W|1|E|Hy|3.37; 501K|1|C|Hn|12.34; 395A|0|E|Hy|7.13; 435I|0|E|Hn|7.14; 508S|1|C|Hy|3.34; 503Q|1|C|Hn|8.37; 494C|0|E|Hn|9.03; 432C|0|C|Hn|15.43 | 510D|1|H|Hy|6.43; 437I|1|E|Hy|4.75; 438L|1|E|Hy|3.63 | 1|1|1; 2|5|4; 3|2|2; 4|6|6; 5|4|5; 6|3|3; 7|8|13; 8|7|8; 9|14|9; 13|9|7 | 10|15|NF; 11|10|NF; 12|11|NF |
| Serine Protease | 213V|0|E|Hn|3.47; 200V|1|E|Hn|12.56; 45S|0|E|Hy|8.20; 192Q|1|C|Hn|3.53; 215W|1|E|Hn|4.08; 228Y|0|E|Hy|6.68; 138I|0|E|Hy|8.15 | 210Q|0|E|Hn|12.74; 226G|0|E|Hn|6.75; 94Y|1|C|Hn|9.22; 78G|1|C|Hy|22.45; 53V|0|E|Hy|7.84 | 1|1|4; 2|4|5; 3|2|3; 4|5|1; 6|7|10; 7|10|12; 8|6|11; 14|8|6 | 10|9|NF; 12|3|NF; 13|11|NF; 15|NF|2; NF|14|12 |
| Smad | 400Q|1|C|Hy|19.87; 337R|1|E|Hn|42.03; 410R|1|C|Hn|20.45; 371A|1|C|Hn|27.39; 289T|1|C|Hn|29.12; 273F|1|C|Hn|34.36; 443N|1|H|Hy|29.70; 278A|1|E|Hy|28.60; 378P|1|C|Hn|46.83; 334R|1|C|Hn|44.99; 354A|1|C|Hy|41.27; 440L|0|E|Hn|29.27; 407Q|1|C|Hy|16.33 | 404A|1|H|Hy|18.41; 392A|1|H|Hy|31.08; 272A|1|C|Hn|36.15 | 1|7|2; 2|8|4; 3|3|1; 4|2|3; 5|9|5; 6|6|7; 7|14|9; 8|1|12; 9|11|11; 10|12|15; 11|13|14; 14|5|6; 15|15|8 | 12|10|NF; 13|NF|10; NF|13|4 |
| Rab56 | 160M|1|E|Hn|10.58; 110R|1|H|Hy|6.14; 171N|1|H|Hy|11.55; 97I|0|E|Hn|9.47; 135A|0|C|Hy|4.90; 111A|0|H|Hy|9.67; 155N|1|C|Hy|15.96; 157L|0|C|Hn|15.00; 148A|0|H|Hy|15.21 | 62V|1|E|Hn|18.43; 82Y|1|C|Hn|3.95; 56A|1|C|Hn|10.94; 168M|1|C|Hn|7.43 | 1|3|2; 2|7|8; 3|2|1; 4|5|9; 5|1|6; 6|4|7; 7|9|4; 8|8|3; 9|11|5 | 13|6|NF; 15|12|NF; 10|NF|12; 14|NF|13 |
| RasRal | 72M|0|H|Hy|12.85; 46I|0|E|Hy|20.61; 25Q|1|H|Hn|11.75; 7V|0|E|Hy|11.07; 24I|1|H|Hy|11.97; 27H|1|C|Hn|8.74; 160V|1|H|Hy|19.53; 65S|1|C|Hy|11.88; 63E|1|C|Hn|8.50; 26N|1|C|Hy|12.38; 6L|0|E|Hn|13.23 | 8V|0|E|Hn|7.25; 51C|1|E|Hn|23.12; 74T|1|H|Hy|17.68 | 1|3|3; 2|9|6; 3|5|2; 4|2|1; 6|6|7; 7|10|12; 8|11|4; 10|4|13; 12|1|5; 13|13|10; 15|8|11 | 9|12|NF; 14|7|NF; 11|NF|9 |

*Three best performing methods, SPEER, GroupSim, MultiRELIEF were employed to identify C3 and C2 sites. Sites are numbered by the following scheme

Residue number (in the representative PDB structure) and type | Solvent accessibility, 0 = buried; 1 = accessible | Secondary structure, H = helix; E = strand; C = coil | Hydrogen bonding, Hy = hydrogen bonded; Hn = not hydrogen bonded | Distance (A°) from specific ligand/protein partner.

# Rank is marked as ‘NF’ (Not Found) wherever the particular subsite is not found within top 15 predictions of any method.
