# Supplementary material for: Ensemble approach to predict specificity determinants: benchmarking and validation
Source: BMC Bioinformatics. 2009 Jul 2;10:207. doi: 10.1186/1471-2105-10-207 (PMC2716344; doi:10.1186/1471-2105-10-207)
Supplement: Additional file 4 — Ensemble approach to predict specificity determinants: benchmarking and validation. Comparison of performance of different methods. [file 1471-2105-10-207-S4.doc]

Additional file 4: Comparison of performance of different methods.

| **Methods** | **Recall (%)** | **Precision (%)** |
| --- | --- | --- |
| **SPEER*** | **44** | **28** |
| **GroupSim*** | **39** | **25** |
| **MultiRELIEF*** | **35** | **23** |
| **SDPpred*** | **37** | **24** |
| **SPEL*** | **37** | **25** |
| **C3_(act+pred)_ sites^#^** | **27^§^** | **35^§^** |
| **C2_(act+pred)_ sites^#^** | **21^§^** | **32^§^** |

***** Top 15 predictions from each method were considered to calculate Recall

and Precision.

**#** C3_(act+pred)_ and C2_(act+pred)_ are commonly predicted sites by SPEER,

GroupSim and MultiRELIEF that include the actual and predicted subsites.

**§** Recall and precision values were calculated using 141 C3_(act+pred)_ and

129 C2_(act+pred)_ sites.
