# Supplementary material for: Ensemble approach to predict specificity determinants: benchmarking and validation
Source: BMC Bioinformatics. 2009 Jul 2;10:207. doi: 10.1186/1471-2105-10-207 (PMC2716344; doi:10.1186/1471-2105-10-207)
Supplement: Additional file 5 — Ensemble approach to predict specificity determinants: benchmarking and validation. Prediction dataset. [file 1471-2105-10-207-S5.doc]

**Additional file 5**: Prediction dataset

| **Family** | **Number of sequences** | **Sequence identity (%)** | **Number of subgroups** | **Representative PDB structure code** |
| --- | --- | --- | --- | --- |
| ADP specific Phosphofructokinase/Glucokinase | 35 | 29 | 7 | 1U2X |
| Protein of unknown function (DUF498orDUF598) | 123 | 27 | 18 | 1IHN |
| Guanine nucleotide exchange factor (GEF)-Ras like GTPases; N terminal domain | 27 | 24 | 7 | 1NVX |
| P21-Rho binding domain | 188 | 27 | 17 | 1CEE |
| Raf-like Ras-binding domain | 145 | 33 | 14 | 1C1Y |
| Ras association (RalGDS/AF-6) domain | 41 | 20 | 9 | 1LFD |
