# Supplementary material for: Ensemble approach to predict specificity determinants: benchmarking and validation
Source: BMC Bioinformatics. 2009 Jul 2;10:207. doi: 10.1186/1471-2105-10-207 (PMC2716344; doi:10.1186/1471-2105-10-207)
Supplement: Additional file 6 — Ensemble approach to predict specificity determinants: benchmarking and validation. List of potential subsites predicted in prediction dataset. [file 1471-2105-10-207-S6.doc]

**Additional file 6: List of potential subsites predicted in prediction dataset.**

| **Family** | **Common in all three methods***  **(C3 sites)** | **Common in any two methods***  **(C2 sites)** | **Ranks of C3 sites^#^**  **SPEER|GroupSim|MultiRELIEF** | **Ranks of C2 sites^#^**  **SPEER|GroupSim|MultiRELIEF** |
| --- | --- | --- | --- | --- |
| ADP specific PhosphofructokiNFse/GlucokiNFse | 138Y|0|E|Hy|; 119Q|1|H|Hy|; 195L|0|E|Hy|; 284A|1|C|Hn| | 183R|0|E|Hy|; 182N|1|E|Hn|; 215E|1|C|Hy|; 309D|1|E|Hy|; 213R|1|C|Hy|; 311A|1|H|Hy|; 210V|0|E|Hn|; 286V|0|C|Hy| | 1|1|6; 5|15|7; 3|12|8 5|15|7; 10|7|3 | 2|2|NF; 4|NF|5; 7|4|NF; 6|NF|2; 8|NF|4; 11|6|NF; 13|3|NF; 15|10|NF |
| Domain of unknown function (DUF498/DUF598) | 98E|1|H|Hy|; 105E|1|H|Hy|; 95P|1|C|Hy|; 107R|1|H|Hy|; 114A|0|E|Hn| | 100I|1|H|Hy|; 118H|1|C|Hy|; 112R|1|C|Hy|; 39V|0|E|Hn| | 1|4|6; 6|1|8; 4|10|9; 5|3|5; 11|8|2 | 3|2|NF; 7|12|NF; 12|9|NF; 15|NF|13 |
| Guanine nucleotide exchange factor (GEF)-Ras like GTPases; N termiNFl domain | 695H|1|H|Hy|; 630T|1|H|Hy|; 691N|1|H|Hy| | 631Y|0|C|Hy|; 643L|1|H|Hy|; 637P|1|H|Hy|; 633S|1|C|Hn|; 697V|1|H|Hy|; 692V|0|H|Hy|; 635C|1|C|Hy|; 648F|1|H|Hy|; 641L|0|H|Hy|; 700H|1|C|Hy|; 699H|1|H|Hn|; 698E|1|H|Hy| | 1|1|1; 2|5|13; 4|4|4 | 5|3|NF; 3|6|NF; 6|7|NF; 7|2|NF; 9|9|NF; 8|NF|6; 10|12|NF; 11|NF|10; 13|13|NF; 14|NF|5; 15|11|NF; NF|9|15 |
| P21-Rho binding domain | 23W|1|C|Hn|; 18V|1|C|Hn|; 19S|1|C|Hn|; 22G|1|C|Hy| | 16K|1|C|Hn|; 21V|1|C|Hn|; 24D|1|C|Hy|; 59K|1|C|Hn|; *52I; 53Y; 54D; 55F; 56I; 57E* | 2|1|3; 3|2|8; 4|3|5; 9|7|15 | 5|5|NF; 1|4|NF; 8|6|NF; 6|8|NF; 12|14|NF; 14|15|NF; 10|11|NF; 13|12|NF; 15|13|NF; 7|9|NF |
| Raf-like Ras-binding domain | 64N|1|C|Hn|; 66Q|1|E|Hn|; 78L|0|H|Hy| | 81C|0|H|Hy|; 69V|1|E|Hn|; 65K|1|C|Hn|; 57T|1|E|Hn|; 67R|1|E|Hn|; 61F|1|E|Hn| | 1|4|3; 7|12|9; 4|10|8 | 3|7|NF; 2|11|NF; 9|1|NF; 6|15|NF; 13|8|NF; 14|2|NF |
| Ras association (RalGDS/AF-6) domain | 63Y|0|E|Hy|; 83V|0|H|Hn|; 43P|1|H|Hy|; 40D|1|C|Hn|; 78P|1|C|Hn| | 20R|1|E|Hn|; 53H|0|C|Hy|; 19I|0|E|Hn|; 80N|1|C|Hn|; 75L|1|E|Hn|; 38S|1|C|Hy|; 85Y|1|H|Hy| | 1|9|1; 2|10|6; 4|1|3; 7|6|11; 8|3|8 | 5|5|NF; 3|11|NF; 11|7|NF; 10|2|NF; 9|NF|9; 13|NF|10; NF|15|4 |

Three best performing methods, SPEER, GroupSim, MultiRELIEF were employed to identify C3 and C2 sites. Sites are numbered by the following scheme

Residue number (in the representative PDB structure) and type | Solvent accessibility, 0 = buried; 1 = accessible | Secondary structure, H = helix; E = strand; C = coil | Hydrogen bonding, Hy = hydrogen bonded; Hn = not hydrogen bonded |. Alignment column that does not have corresponding residue in representative structure is shown in italics.

Rank is marked as ‘NF’ (Not Found) wherever the particular subsite is not found within top 15 predictions of any method.
