# Supplementary material for: Ensemble approach to predict specificity determinants: benchmarking and validation
Source: BMC Bioinformatics. 2009 Jul 2;10:207. doi: 10.1186/1471-2105-10-207 (PMC2716344; doi:10.1186/1471-2105-10-207)
Supplement: Additional file 7 — Ensemble approach to predict specificity determinants: benchmarking and validation. Spatial distances among the predicted C3 and C2 sites for six families within the prediction dataset. [file 1471-2105-10-207-S7.doc]

Additional file 7

**
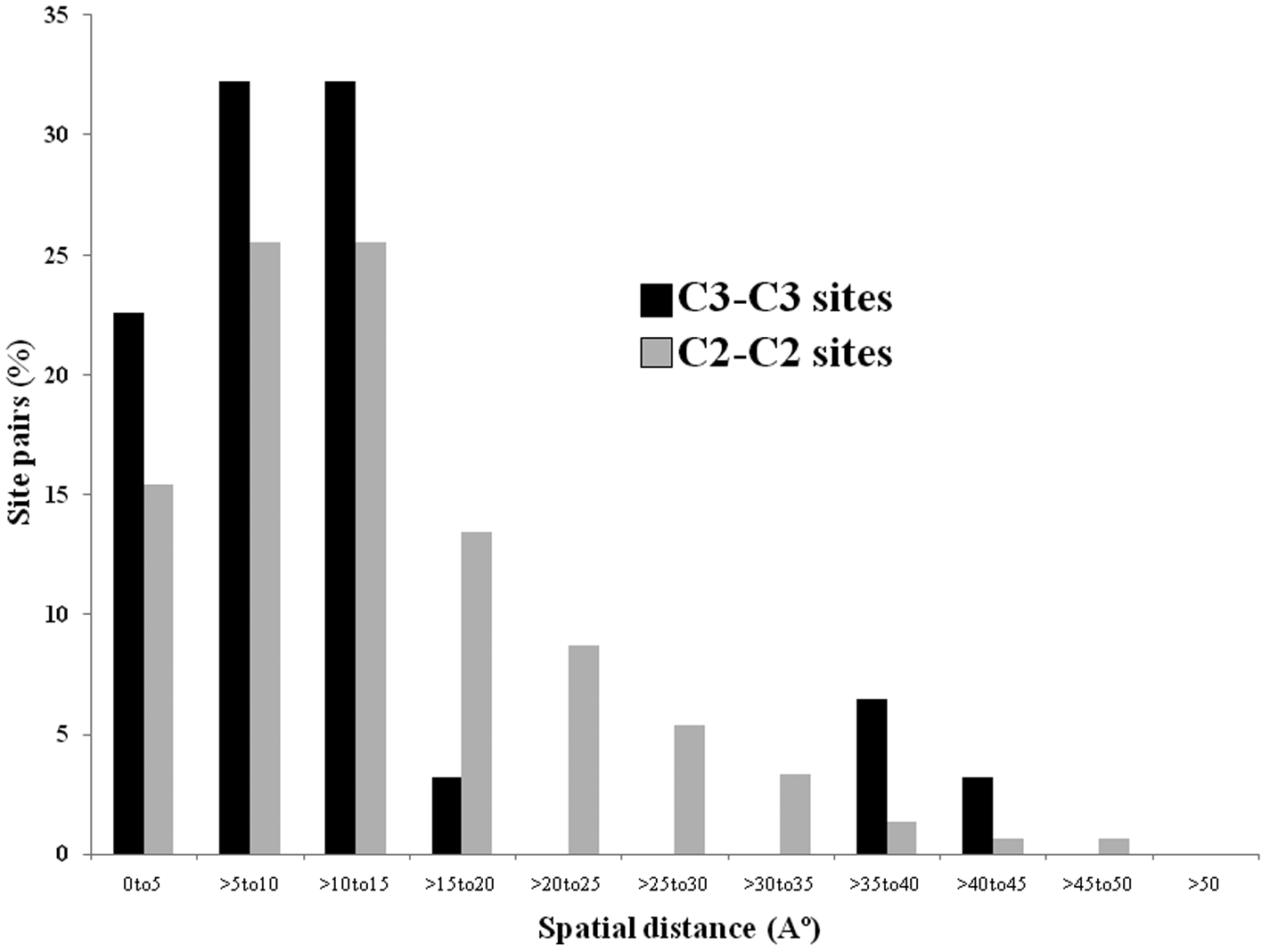
**

Spatial distances among the predicted C3 and C2 sites for six families within the prediction dataset. Minimum atom distances were calculated to estimate spatial distances between two protein residues. Black and grey bars represent the distances within the C3 and C2 sites, respectively.
