# Supplementary material for: Ensemble approach to predict specificity determinants: benchmarking and validation
Source: BMC Bioinformatics. 2009 Jul 2;10:207. doi: 10.1186/1471-2105-10-207 (PMC2716344; doi:10.1186/1471-2105-10-207)
Supplement: Additional file 8 — Ensemble approach to predict specificity determinants: benchmarking and validation. Structural property analysis of potential (C3 and C2 sites) subsites within the prediction dataset. [file 1471-2105-10-207-S8.doc]

Additional file 8

**
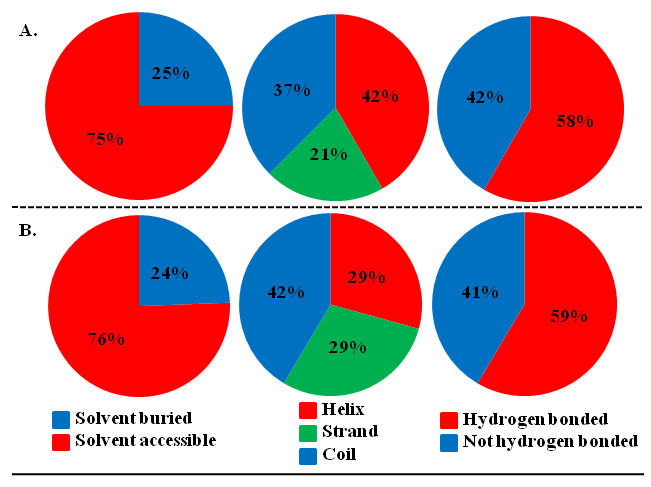
**

Structural property analysis of potential (C3 and C2 sites) subsites within the prediction dataset. Solvent accessibility, secondary structural content and hydrogen bonding patterns for C3 (A) and C2 sites (B) were computed from the individual protein structure using the JOY package.
