# Supplementary material for: Ensemble approach to predict specificity determinants: benchmarking and validation
Source: BMC Bioinformatics. 2009 Jul 2;10:207. doi: 10.1186/1471-2105-10-207 (PMC2716344; doi:10.1186/1471-2105-10-207)
Supplement: Additional file 9 — Ensemble approach to predict specificity determinants: benchmarking and validation. Validation dataset. [file 1471-2105-10-207-S9.doc]

**Additional file 9**: Validation dataset

| **Code** | **Description** | **No. of subgroup** | **No. of Family member** | **Representative PDB structure code** | **No. of subsites** | **Structural Class and Fold** | **Sequence identity (%)** | **RMSD** | **Alignment**  **length** | **Source of evidences on subsites** | **References** |
| --- | --- | --- | --- | --- | --- | --- | --- | --- | --- | --- | --- |
| cd00120 | MADS: MCM1, Agamous, Deficiens, and SRF box family. | 2 | 90 | 1EGW_A | 3 | α + β; SRF-like | 12 | 0.9 | 1108 | Experimental | (1,2) |
| cd00264 | Bactericidal permeability-increasing protein, lipopolysaccharide-binding protein and cholesteryl ester transfer protein domains | 2 | 31 | 1BP1_1 | 3 | α + β; BPI domain like | 8 | -- | 831 | Structural and computational | (3-6) |
| cd00333 | Major intrinsic protein (MIP) family | 2 | 27 | 1FX8_A | 12 | membrane proteins; Aquaporin | 20 | 2.2 | 1118 | Experimental, structural and computational | (7-10) |
| cd00363 | Phosphofructokinase | 2 | 11 | 1PFK_A | 6 | α / β; Phosphofructokinase | 35 | 0.9 | 1101 | Experimental | (11) |
| cd00365 | Hydroxymethylglutaryl-coenzyme A (HMG-CoA) reductase | 2 | 30 | 1DQA | 10 | α + β;  Substrate-binding domain of HMG-CoA reductase | 24 | 1.8 | 1151 | Experimental | (12, 13) |
| cd00423 | Pterin binding enzymes | 2 | 33 | 1AJ0 | 4 | α / β; TIM barrel | 16 | 2.4 | 1394 | Experimental | (14-16) |
| cd00985 | Maf_Ham1 family | 2 | 180 | 2MJP_A | 3 | α / β; Anticodon binding domain-like | 17 | 2.1 | 1051 | Experimental | (17-18) |
| Gprotein | G protein alpha subunit | 11 | 105 | 1FQJ | 7 | Α; Transducin | 47 | 0.8 | 310 | Experimental, structural and computational | (7,8,19) |
| GST | Glutathione S-transferase family | 11 | 107 | 2GST | 9 | α / β (N term.), α (C term.); GST | 20 | 2.5 | 330 | Experimental | (19, 20) |
| LacI | LacI/PurR family | 15 | 54 | 1WET | 12 | α (N term.), α / β (C term.); lambda repressor-like; Periplasmic binding protein-like | 27 | 0.2 | 340 | Experimental, structural and computational | (7,8,19,21, 22) |
| Ricin | RICIN domain family | 3 | 47 | 1ISY | 21 | Β; beta-trefoil | 37 | 1.3 | 135 | Experimental | (23,24) |
| CBM9 | Family 9 carbohydrate-binding module | 2 | 19 | 1I82 | 7 | Β; Immunoglobin-like beta sandwitch | 37 | 0.1 | 196 | Experimental | (25) |
| IDH_IMDH | Isocitrate /Isopropylmalate dehydrogenase | 2 | 68 | 1AI2 | 14 | α / β; IDH/IMDH | 35 | 2.1 | 745 | Experimental | (26-31) |
| Serine protease | Serine protease | 3 | 96 | 5PTP | 2 | Β; Trypsin-like serine protease | 47 | 0.6 | 284 | Experimental | (32) |
| Nucleotidyl cyclase | Nucleotidyl cyclase | 2 | 50 | 1CS4 | 2 | α + β; Ferredoxcin-like | 47 | 0.6 | 231 | Experimental | (32-33) |
| LDH_MDH | Lactate/ Malate dehydrogenase | 2 | 44 | 9LDT | 1 | α / β; NADP binding Rossmann | 36 | 0.9 | 180 | Experimental | (32, 34-35) |
| CNmyc | C and N terminal Myc | 2 | 34 | -- | 11 | -- | 33 | -- | 583 | Computational | (36) |
| Smad | Smad family of TGF β associated transcription factors | 5 | 33 | 1KHX | 28 | Β; SMAD/FHA domain | 76 | 1.0 | 211 | Experimental | (37-40) |
| Rab56 | Rab 5 and Rab 6 subfamilies of Ras family | 2 | 10 | 2HEI | 28 | α / β; Rossmann | 49 | 1.6 | 162 | Experimental | (37, 38,41) |
| RasRal | Ras and Ral subfamilies of Ras family | 2 | 93 | 521P | 12 | α / β; P-loop containing nucleoside triphosphate hydrolases | 55 | 0.7 | 217 | Experimental | (37, 38-41) |

**Reference:**

1. Santelli E, Richmond TJ. Crystal structure of MEF2A core bound to DNF at 1.5A resolution. *J Mol Biol* 2000; **297**: 437-449.
2. Tan S, Richmond TJ. Crystal structure of the yeast MATalpha2/MCM1/DNF ternary complex. *Nature* 1998; **391**: 660-666.
3. Ooi CE, Weiss J, Elsbach P, Frangione B, Mannion B. A 25-kDa NH2-termiNFl fragment carries all the antibacterial activities of the human neutrophil 60-kDa bactericidal/permeability-increasing protein. *J Biol Chem* 1987; **262**: 14891-14894.
4. Ooi CE, Weiss J, Doerfler ME, Elsbach P. Endotoxin-neutralizing properties of the 25 kD N-terminal fragment and a newly isolated 30 kD C-terminal fragment of the 55-60 kD bactericidal/permeability-increasing protein of human neutrophils. *J Exp Med* 1991; **174**: 649-655.
5. Abrahamson SL, Wu HM, Williams RE, Der K, Ottah N, Little R, Gazzano-Santoro, H, Theofan G, Bauer R, Leigh S, Orme A, Horwitz AH, Carroll SF, Dedrick RL. Biochemical characterization of recombinant fusions of lipopolysaccharide binding protein and bactericidal/permeability-increasing protein. Implications in biological activity. *J Biol Chem* 1997; **272**: 2149-2155.
6. Beamer LJ, Carroll SF, Eisenberg D. Crystal Structure of Human BPI and Two Bound Phospholipids at 2.4 Angstrom Resolution. *Science* 1997; **276**: 1861-1864.
7. Kalinina OV, Novichkov PS, Mironov AA, Gelfand MS, Rakhmaninova AB. SDPpred: a tool for prediction of amino acid residues that determine differences in functional specificity of homologous proteins. *Nucleic Acids Res* 2004; **32**: W424-428.
8. Kalinina OV, Mironov AA, Gelfand MS, Rakhmaninova AB. Automated selection of positions determining functional specificity of proteins by comparative analysis of orthologous groups in protein families. *Protein Sci* 2004; **13**: 443-456.
9. Fu D, Libson A, Miercke LJ, Weitzman C, Nollert P, Krucinski J, Stroud RM. Structure of a glycerol-conducting channel and the basis for its selectivity. *Science* 2000; **290**: 481–486.
10. Sui H, Han BG, Lee JK, Walian P, Jap BK. Structural basis of water-specific transport through the AQP1 water channel. *Nature* 2001; **414**: 872–878.
11. Moor SA, Ronimus RS, Roberson RS, Morgan HW. The Structure of a Pyrophosphate-Dependent Phosphofructokinase from the Lyme Disease Spirochete *Borrelia burgdorfer.* *Structure* 2002; **10**: 659–671.
12. Bochar DA, Stauffacher CV, Rodwell VW. Sequence Comparisons Reveal Two Classes of 3-Hydroxy-3-methylglutaryl Coenzyme A Reductase. *Molecular Genetics and Metabolism* 1999; **66**: 122–127.
13. Istvan ES. Bacterial and mammalian HMG-CoA reductases: related enzymes with distinct architectures. *Curr Opin Struct Biol* 2001; **11**:746-751.
14. Hampele IC, D'Arcy A, Dale GE, Kostrewa D, Nielsen J, Oefner C, Page MG, Schonfeld HJ, Stuber D, Then RL. Structure and function of the dihydropteroate synthase from *Staphylococcus aureus*. *J Mol Biol* 1997; **268**: 21-30.
15. Achari A, Somers DO, Champness JN, Bryant PK, Rosemond J, Stammers DK. Crystal structure of the anti-bacterial sulfonamide drug target dihydropteroate synthase. *Nat Struct Biol* 1997; **4**: 490-497.
16. Doukov T, Seravalli J, Stezowski JJ, Ragsdale SW. Crystal structure of a methyltetrahydrofolate- and corrinoiddependent methyltransferase. *Structure* 2000; **8**: 817–830
17. Minasov G, Teplova M, Stewart GC, Koonin EV, Anderson WF, Egli M. Functional implications from crystal structures of the conserved *Bacillus subtilis* protein Maf with and without dUTP. *Proc Natl Acad Sci U S A* 2000; **97**: 6328–6333.
18. Hwang KY, Chung JH, Kim SH, Han YS, Cho Y. Structure-based identification of a novel NTPase from *Methanococcus jannaschii. Nat Struct Biol* 1999; **6**: 691-696.
19. Pei J, Cai W, Kinch LN, Grishin NV. Prediction of functional specificity determinants from protein sequences using log-likelihood ratios. *Bioinformatics* 2006; **22**: 164-171.
20. Sheehan D, Meade G, Foley VM, Dowd CA. Structure, function and evolution of glutathione transferases: implications for classification of non-mammalian members of an ancient enzyme superfamily. *Biochem J* 2001; **360**: 1-16.
21. Mirny LA, Gelfand MS. Using orthologous and paralogous proteins to identify specificity-determining residues in bacterial transcription factors. *J Mol Biol* 2002; **321**: 7-20.
22. Suckow J, Markiewicz P, Kleina LG, Miller J, Kisters-Woike B, Müller-Hill BGenetic studies of the Lac repressor. XV: 4000 single amino acid substitutions and analysis of the resulting phenotypes on the basis of the protein structure. *J Mol Biol* 1996; **261**: 509-523.
23. Rutenber E, Ready M, Robertus JD. Structure and evolution of ricin B chain. *Nature* 1987; **326**: 624-626.
24. Pils B, Copley RC, Schultz J. Variation in structural location and amino acid conservation of functional sites in protein domain families. *BMC Bioinformatics* 2005; **6**: 210-219.
25. Notenboom V, Boraston AB, Kilburn DG, Rose DR. Crystal Structures of the Family 9 Carbohydrate-Binding Module from *Thermotoga maritima* Xylanase 10A in native and Ligand-Bound Forms. *Biochemistry* 2001; **40**: 6248-6256.
26. Kalinina O.V., Gelfand M.S. Amino Acid Residues that Determine Functional Specificity of NADP- and NAD-Dependent Isocitrate and Isopropylmalate Dehydrogenases. *Proteins.* 2006, **64**:1001–1009.
27. Xu X., Zhao J., Peng B., Huang Q., Arnold E., Ding J. Structure of human cytosolic NADP-dependent dehydrogenase reveals a novel self-regulatory mechanism of activity. *J Biol Chem.* 2004, **279**:22946–33957.
28. Miyazaki K., Yaoi T., Oshima T. Expression, purification, and substrate specificity of isocitrate dehydrogenase from *Thermus* *thermophilus* HB8. *Eur J Biochem*. 1994., **221**:899–903.
29. Doyle S.A., Beernink P.T., Koshland D.E. Jr. Structural basis for a change in substrate specificity: crystal structure of S113E isocitrate dehydrogenase in a complex with isoporpylmalate, Mg2+ and NFDP. *Biochemistry* 2001, **40** :4234–4241.
30. Zhang T. Koshland D.E. Jr. Modeling substrate binding in *Thermus thermophilus* isopropylmalate dehydrogenase. *Protein Sci.* 1995, **4**:84–92.
31. Zhu G., Golding G.B., Dean A.M. The selective cause of an ancient adaptation. *Science* 2005, **307**:1279–1282.
32. Wallace I.M. Higgins D.G. Supervised multivariate analysis of sequence groups to identify specificity determining residues. *BMC Bioinformatics* 2007, **8**:135-146.
33. Tucker CL, Hurley JH, Miller TR, Hurley JB. Two amino acid substitutions convert a guanylyl cyclase, RetGC-1, into an adenylyl cyclase. *Proc Natl Acad Sci U S A*. 1998, **95**: 5993-5997.
34. Wilks HM, Hart KW, Feeney R, Dunn CR, Muirhead H, Chia WN, Barstow DA, Atkinson T, Clarke AR, Holbrook JJ. A specific, highly active malate dehydrogenase by redesign of a lactate dehydrogenase framework. *Science* 1988, **242**:1541-1544.
35. Pazos F, Rausell A, Valencia A. Phylogeny-independent detection of functional residues. *Bioinformatics* 2006, **22**:1440-1448.
36. Knudsen, B & Miyamoto, M.M A likelihood ratio test for evolutionary rate shifts and functional divergence among proteins. *Proc Natl Acad Sci U S A* 2001, **98**: 14512-14517.
37. Ye K, Anton Feenstra K, Heringa J, Ijzerman AP, Marchiori E: Multi-RELIEF: a method to recognize specificity determining residues from multiple sequence alignments using a Machine-Learning approach for feature weighting. *Bioinformatics* 2008, **24**:18-25.
38. Pirovano W, Feenstra KA, Heringa J: Sequence comparison by sequence harmony identifies subtype-specific functional sites. *Nucleic Acids Res*. 2006, **34**: 6540-6548.
39. Feng,X. and Derynck,R. Specificity and versatility in TGF-beta signaling through Smads. *Annu. Rev. Cell Dev. Biol*., 2005, **21**: 659–693.
40. Massagué J, Seoane J, Wotton D. Smad transcription factors. *Genes Dev*. **19**: 2783–2810.
41. Reuther,G. and Der,C. The Ras branch of small GTPases: Ras family members don’t fall far from the tree. *Curr. Opin. Cell Biol*. 2000, **12**: 157–165.
