# Supplementary material for: Ensemble approach to predict specificity determinants: benchmarking and validation
Source: BMC Bioinformatics. 2009 Jul 2;10:207. doi: 10.1186/1471-2105-10-207 (PMC2716344; doi:10.1186/1471-2105-10-207)
Supplement: Additional file 10 — Ensemble approach to predict specificity determinants: benchmarking and validation. List of actual subsites. [file 1471-2105-10-207-S10.doc]

**Additional file 10: List of actual specificity determining sites.**

| **Family code** | **Sites*** | **Ligand(s)** | **Distance from ligand(s)** | **Family code** | **Sites** | **Ligand(s)** | **Distance from ligand(s)** |
| --- | --- | --- | --- | --- | --- | --- | --- |
| GST | 6Y|0|E|Hn| | 9-(s-glutathionyl)-10-hydroxy-9,10- dihydrophenanthrene (**GPS**) | 3.24 | ricin | 411G|1|C|Hn| | Glucose (**GLC**) | 2.94 |
| GST | 7W\|0\|E\|Hy\| |  | 3.28 | ricin | 423Y\|1\|E\|Hy\| |  | 3.55 |
| GST | 9V\|1\|C\|Hn\| |  | 3.61 | ricin | 432R\|1\|C\|Hy\| |  | 8.98 |
| GST | 11G\|0\|C\|Hy\| |  | 3.45 | ricin | 434T\|1\|E\|Hn\| |  | 15.63 |
| GST | 12L\|1\|C\|Hn\| |  | 3.65 | ricin | 435R\|1\|C\|Hn\| |  | 16.63 |
| GST | 22Y\|1\|H\|Hy\| |  | 21.01 | ricin | 436T\|1\|C\|Hy\| |  | 12.87 |
| GST | 23T\|1\|C\|Hy\| |  | 21.25 | IDH_IMDH | 100K\|0\|E\|Hy\| | Isocitrate calcium complex  (**ICA**)  Nicotinamide-adenine-dinucleotide phosphate  (**NADP**) | 3.68 |
| GST | 102Q\|1\|H\|Hy\| |  | 12.47 | IDH_IMDH | 105T\|1\|C\|Hn\| |  | 4 |
| GST | 115Y\|1\|C\|Hy\| |  | 3.33 | IDH_IMDH | 115N\|0\|H\|Hy\| |  | 2.51 |
| Gprotein | 46V\|1\|H\|Hy\| | Regulator of G-protein signaling 9 (**RGS**);  phosphodiesterase gamma-subunit (**PGE**) | 8.49 | IDH_IMDH | 135Q\|1\|C\|Hn\| |  | 15.72 |
| Gprotein | 145N\|1\|C\|Hy\| |  | 9.95 | IDH_IMDH | 155N\|0\|C\|Hy\| |  | 6.68 |
| Gprotein | 236M\|1\|C\|Hn\| |  | 4.11 | IDH_IMDH | 161A\|1\|C\|Hy\| |  | 9.04 |
| Gprotein | 244K\|1\|H\|Hy\| |  | 2.96 | IDH_IMDH | 232N\|1\|C\|Hn\| |  | 22.59 |
| Gprotein | 250C\|0\|H\|Hn\| |  | 7.51 | IDH_IMDH | 233I\|1\|C\|Hn\| |  | 21.5 |
| Gprotein | 252N\|1\|C\|Hn\| |  | 3.1 | IDH_IMDH | 292R\|1\|C\|Hy\| |  | 21.79 |
| Gprotein | 336T\|0\|H\|Hy\| |  | 20.63 | IDH_IMDH | 327N\|0\|E\|Hy\| |  | 6.7 |
| LacI | 15T\|1\|H\|Hn\| | Deoxy-ribonucleic acid (**DNA**)  Guanine (**GUN**) | 4.81 | IDH_IMDH | 341T\|0\|C\|Hn\| |  | 2.5 |
| LacI | 16T\|1\|H\|Hy\| |  | 3.46 | IDH_IMDH | 344K\|1\|C\|Hn\| |  | 3.78 |
| LacI | 50V\|1\|H\|Hy\| |  | 6.93 | IDH_IMDH | 345Y\|1\|C\|Hn\| |  | 2.6 |
| LacI | 55K\|1\|H\|Hy\| |  | 3.27 | IDH_IMDH | 351V\|0\|C\|Hn\| |  | 3.31 |
| LacI | 85C\|0\|H\|Hy\| |  | 20.59 | MDH_LDH | 102Q\|1\|C\|Hn\| |  | 4.18 |
| LacI | 98W\|1\|C\|Hy\| |  | 9.3 | nucleotidyl_cyclase | 505D\|1\|E\|Hn\| | Foskolin (**FOK**) | 4.53 |
| LacI | 114K\|1\|H\|Hy\| |  | 10.04 | nucleotidyl_cyclase | 436K\|1\|E\|Hn\| |  | 6.41 |
| LacI | 122M\|0\|C\|Hn\| |  | 7.87 | serine | 189D\|0\|C\|Hn\| | Monoisopropylphosphorylserine (**MIS**) | 7.17 |
| LacI | 123C\|0\|C\|Hy\| |  | 7.45 | serine | 221A\|0\|C\|Hy\| |  | 8.54 |
| LacI | 146D\|1\|C\|Hn\| |  | 7.3 | Smad | 267T\|1\|C\|Hn\| | Phosphonoserine (**SEP**) | 30.38 |
| LacI | 147W\|0\|C\|Hn\| |  | 10.06 | Smad | 284Q\|1\|E\|Hn\| |  | 26.49 |
| LacI | 160D\|1\|C\|Hy\| |  | 6.89 | Smad | 294Q\|1\|C\|Hy\| |  | 41.45 |
| LacI | 221F\|1\|C\|Hy\| |  | 3.23 | Smad | 295P\|1\|C\|Hy\| |  | 42.52 |
| LacI | 249I\|1\|H\|Hy\| |  | 5.96 | Smad | 297L\|0\|E\|Hn\| |  | 39.18 |
| cbm9 | 71W\|1\|C\|Hn\| | Glucose (**GLC**) | 3.75 | Smad | 298T\|1\|E\|Hn\| |  | 41.81 |
| cbm9 | 77E\|0\|E\|Hy\| |  | 2.69 | Smad | 308S\|1\|C\|Hn\| |  | 42.35 |
| cbm9 | 96Q\|0\|E\|Hy\| |  | 2.61 | Smad | 309E\|1\|C\|Hn\| |  | 43.84 |
| cbm9 | 98R\|0\|E\|Hy\| |  | 3 | Smad | 323A\|1\|H\|Hn\| |  | 38.57 |
| cbm9 | 151Q\|0\|E\|Hy\| |  | 2.97 | Smad | 325V\|0\|H\|Hy\| |  | 34.64 |
| cbm9 | 172N\|1\|C\|Hy\| |  | 2.35 | Smad | 327M\|1\|H\|Hy\| |  | 41.44 |
| cbm9 | 175W\|1\|C\|Hy\| |  | 3.39 | Smad | 341I\|1\|E\|Hn\| |  | 42.75 |
| cd00120 | (48F)\|1\|E\|Hn\| | No ligand | -- | Smad | 346F\|1\|E\|Hn\| |  | 39.95 |
| cd00120 | (56Q)\|1\|E\|Hn\| |  | -- | Smad | 360P\|1\|H\|Hn\| |  | 27.27 |
| cd00120 | (59S)\|1\|C\|Hy\| |  | -- | Smad | 364Q\|1\|H\|Hn\| |  | 29.03 |
| cd00264 | 8R\|1\|E\|Hy\| | Phosphatidylcholine (**PC**) | 6.54 | Smad | 365R\|1\|H\|Hy\| |  | 34.47 |
| cd00264 | 14L\|0\|H\|Hy\| |  | 4.32 | Smad | 366Y\|1\|C\|Hy\| |  | 36.6 |
| cd00264 | 21G\|0\|H\|Hy\| |  | 3.43 | Smad | 368W\|1\|C\|Hy\| |  | 31.96 |
| cd00333 | 20L\|1\|H\|Hy\| | Glycerol (**CRY**) | 9.42 | Smad | 381N\|1\|E\|Hn\| |  | 43.65 |
| cd00333 | 24F\|1\|H\|Hy\| |  | 8.68 | Smad | 427R\|1\|C\|Hy\| |  | 33.07 |
| cd00333 | 43E\|1\|H\|Hy\| |  | 9.96 | Smad | 430T\|1\|C\|Hn\| |  | 25.66 |
| cd00333 | 48W\|0\|H\|Hy\| |  | 3.13 | Smad | 460S\|1\|C\|Hn\| |  | 10.11 |
| cd00333 | 108Y\|1\|H\|Hy\| |  | 15.07 | Smad | 461V\|1\|C\|Hn\| |  | 7.08 |
| cd00333 | 187I\|0\|H\|Hy\| |  | 3.69 | Smad | 462R\|1\|C\|Hn\| |  | 4.09 |
| cd00333 | 191G\|0\|H\|Hy\| |  | 7.01 | Smad | 463C\|1\|C\|Hn\| |  | 3.31 |
| cd00333 | 200F\|0\|C\|Hn\| |  | 2.91 | Smad | 466M\|1\|C\|Hn\| |  | 1.32 |
| cd00333 | 201A\|0\|C\|Hn\| |  | 3.31 | Rab56 | 42K\|1\|H\|Hy\| | (4S,5S)-1,2-dithiane-4,5-diol  (**D1D**)  Guanosine-5'-diphosphate  (**GDP**) | 12.52 |
| cd00333 | 207D\|0\|H\|Hy\| |  | 6.59 | Rab56 | 43G\|1\|C\|Hy\| |  | 12.18 |
| cd00333 | 211K\|0\|H\|Hy\| |  | 9.34 | Rab56 | 44Q\|1\|C\|Hy\| |  | 9.12 |
| cd00333 | 236P\|0\|H\|Hn\| |  | 8.35 | Rab56 | 46H\|1\|C\|Hy\| |  | 4.23 |
| cd00363 | 41Y\|0\|H\|Hy\| | Beta-fructose-1,6-diphosphate (**FBP**)  adenosine-5'-diphosphate (**ADP**) | 3.5 | Rab56 | 47E\|1\|C\|Hn\| |  | 3.58 |
| cd00363 | 104G\|1\|H\|Hy\| |  | 3.23 | Rab56 | 48Y\|1\|C\|Hn\| |  | 3.41 |
| cd00363 | 107M\|1\|H\|Hy\| |  | 3.67 | Rab56 | 49Q\|1\|C\|Hn\| |  | 4.68 |
| cd00363 | 108G\|0\|H\|Hy\| |  | 3.59 | Rab56 | 50E\|1\|C\|Hy\| |  | 4.81 |
| cd00363 | 162R\|1\|C\|Hy\| |  | 8.26 | Rab56 | 51S\|1\|C\|Hn\| |  | 2.47 |
| cd00363 | 249H\|1\|C\|Hn\| |  | 3.25 | Rab56 | 83H\|1\|C\|Hy\| |  | 2.73 |
| cd00365 | 565S\|0\|H\|Hy\| | Coenzyme a (**CoA**)  3-hydroxy-3-methyl-glutaric acid (**MAH**)  Nicotinamide-adenine-dinucleotide phosphate  (**NADP**) | 2.74 | Rab56 | 86A\|0\|H\|Hn\| |  | 3.54 |
| cd00365 | 567N\|1\|H\|Hy\| |  | 2.99 | Rab56 | 88M\|1\|H\|Hy\| |  | 6.64 |
| cd00365 | 568R\|0\|H\|Hy\| |  | 2.96 | Rab56 | 90Y\|0\|H\|Hn\| |  | 3.53 |
| cd00365 | 571R\|1\|H\|Hy\| |  | 3.32 | Rab56 | 92G\|1\|C\|Hy\| |  | 11.67 |
| cd00365 | 752H\|0\|C\|Hy\| |  | 3.68 | Rab56 | 93A\|0\|C\|Hn\| |  | 9.6 |
| cd00365 | 849G\|0\|H\|Hy\| |  | 5.13 | Rab56 | 94Q\|1\|C\|Hy\| |  | 12.86 |
| cd00365 | 852S\|0\|H\|Hy\| |  | 2.85 | Rab56 | 117E\|1\|H\|Hy\| |  | 2.58 |
| cd00365 | 853L\|0\|H\|Hy\| |  | 3.67 | Rab56 | 118L\|0\|H\|Hy\| |  | 7.43 |
| cd00365 | 865S\|1\|H\|Hy\| |  | 3.27 | Rab56 | 119Q\|1\|H\|Hy\| |  | 10.31 |
| cd00365 | 869H\|1\|H\|Hn\| |  | 3.71 | Rab56 | 120R\|1\|H\|Hy\| |  | 8.84 |
| cd00423 | 220R\|1\|C\|Hy\| | Sulfanilamide (**SAN**) | 2.98 | Rab56 | 121Q\|1\|H\|Hy\| |  | 6.47 |
| cd00423 | 221K\|0\|C\|Hn\| |  | 3.42 | Rab56 | 122A\|0\|C\|Hn\| |  | 9.22 |
| cd00423 | 255R\|0\|E\|Hy\| |  | 5.82 | Rab56 | 123S\|1\|C\|Hn\| |  | 11.71 |
| cd00423 | 257H\|1\|C\|Hy\| |  | 3.67 | Rab56 | 124P\|1\|C\|Hn\| |  | 14.93 |
| cd00985 | 15T\|1\|C\|Hn\| | Phosphoaminophosphonic acid-adenylate ester (**ANP**) | 7.94 | Rab56 | 125S\|1\|C\|Hn\| |  | 16.3 |
| cd00985 | 17N\|1\|C\|Hy\| |  | 2.5 | Rab56 | 126I\|0\|C\|Hn\| |  | 10.39 |
| cd00985 | 20K\|1\|H\|Hy\| |  | 3.33 | Rab56 | 127V\|1\|E\|Hn\| |  | 13.89 |
| ricin | 313G\|1\|C\|Hy\| | Glucose (**GLC**) | 12.71 | Rab56 | 183K\|1\|C\|Hn\| |  | 25.19 |
| ricin | 325D\|0\|E\|Hy\| |  | 2.56 | RasRal | 21I\|1\|H\|Hy\| | Guanosine-5'-triphosphate  (**GTP**) | 6.75 |
| ricin | 338Q\|1\|E\|Hn\| |  | 3.1 | RasRal | 22Q\|1\|H\|Hy\| |  | 7.81 |
| ricin | 340Y\|1\|E\|Hy\| |  | 3.29 | RasRal | 30D\|1\|C\|Hn\| |  | 3 |
| ricin | 346T\|1\|C\|Hy\| |  | 7.68 | RasRal | 31E\|1\|C\|Hn\| |  | 3.98 |
| ricin | 348Q\|0\|C\|Hy\| |  | 4.47 | RasRal | 33D\|1\|C\|Hn\| |  | 4.5 |
| ricin | 354D\|1\|C\|Hn\| |  | 24.75 | RasRal | 36I\|1\|C\|Hn\| |  | 5.91 |
| ricin | 355A\|1\|C\|Hy\| |  | 24.75 | RasRal | 37E\|1\|E\|Hn\| |  | 7.6 |
| ricin | 360V\|0\|E\|Hy\| |  | 10.17 | RasRal | 43Q\|1\|E\|Hy\| |  | 20.88 |
| ricin | 364K\|1\|E\|Hy\| |  | 13.72 | RasRal | 53L\|0\|E\|Hn\| |  | 17.2 |
| ricin | 368A\|0\|E\|Hy\| |  | 14.88 | RasRal | 67M\|1\|C\|Hy\| |  | 13.76 |
| ricin | 369A\|1\|C\|Hn\| |  | 14.97 | RasRal | 70Q\|1\|H\|Hy\| |  | 16.81 |
| ricin | 370G\|1\|C\|Hy\| |  | 14.79 | RasRal | 92D\|1\|C\|Hy\| |  | 10.46 |
| ricin | 408D\|0\|E\|Hy\| |  | 2.43 | RasRal | 67M\|1\|C\|Hy\| |  | 13.76 |
| ricin | 410V\|1\|C\|Hn\| |  | 3.69 | RasRal | 70Q\|1\|H\|Hy\| |  | 16.81 |
|  |  |  |  | RasRal | 92D\|1\|C\|Hy\| |  | 10.46 |

*Residue number (in the representative PDB structure) and type | Solvent accessibility, 0 = buried; 1 = accessible | Secondary structure, H = helix, E = strand; C = coil | Hydrogen bonding, Hy = hydrogen bonded; Hn = not hydrogen bonded.
