# Supplementary material for: Ensemble approach to predict specificity determinants: benchmarking and validation
Source: BMC Bioinformatics. 2009 Jul 2;10:207. doi: 10.1186/1471-2105-10-207 (PMC2716344; doi:10.1186/1471-2105-10-207)
Supplement: Additional file 11 — Ensemble approach to predict specificity determinants: benchmarking and validation. Additional information about validation dataset. [file 1471-2105-10-207-S11.doc]

Additional file 11

*cd00120:*

MCM1, Agamous, Deficiens, and SRF (serum response factor) box family (cd00120) of eukaryotic transcriptional regulators form the MADS family. These proteins bind DNA and exist as hetero and homo-dimers. This family is composed of 2 main subfamilies: SRF-like/Type I and MEF2-like (myocyte enhancer factor 2)/ Type II as suggested by CDD database. Apart from the extra alpha 2 helix responsible for the dimerization interface in SRF-like/Type I subfamily there are three other sites that could be important in specificity determination. Two of these sites (see Table SM3) were identified as a phosphorylation sites in the MADS_MEF2-like subfamily and were linked to increased DNA binding affinity (1, 2). The third one is a part of the dimerization interface.

*cd00264:*

Bactericidal permeability-increasing (BPI) proteins (cd00264) bind to and neutralize lipopolysaccharides (LPS) from the outer membrane of Gram-negative bacteria. Apolar pockets, formed mainly by helix A on the concave surface bind a molecule of phosphatidylcholine, primarily by interacting with their acyl chains. It consists of two domains of similar sizes (N-terminal BPI1 and C-terminal BPI2) that are connected by a proline rich linker of 21 residues (positions 230 to 250).The N-terminal domain of BPI is cationic and retains the bactericidal, LPS binding, and LPS-neutralization activities of the intact protein (3,4). The COOH-terminal domain is essentially neutral and shows limited LPS-neutralization activity (5). From the study of Beamer *et al*., 1997 (6) we extrapolated that three sites (Arg8, Leu14 and Gly21 in BPI1) residing just before and within the helix A and A` (in BPI1 and BPI2, respectively) could be responsible for variable bactericidal binding properties and may be important for subfamily specificity.

*cd00333:*

The major intrinsic protein (MIP) family (cd00333) is a large and diverse family of transmembrane channels containing two major subfamilies with bacterial members: glycerol-transporting channel proteins (GLP) and aquaporins (AQPs), water-transporting channel proteins. For the current study 12 sites were selected as specificity determinant involving in either pore selectivity, water channel formation in AQPs or interaction with glycerol in GLPs (7- 10).

*cd00363:*

Phosphofructokinase (PFK; cd00363) catalyzes the phosphorylation of fructose-6-phosphate to fructose-1,6-biphosphate. PFK family contains two subfamilies; ATP and pyrophosphate (PPi) dependent phosphofructokinases. Generally, ATP-PFKs are allosteric homotetramers, and PPi-PFKs are dimeric and nonallosteric except for plant PPi-PFKs which are allosteric heterotetramers. Six sites that have been suggested to be important in maintaining specific binding to ATP or PPi were selected as true positives for this analysis (11).

*cd00365:*

Hydroxymethylglutaryl-coenzyme A (HMG-CoA) reductase (HMGR; cd00365) is a tightly regulated enzyme, which catalyzes the synthesis of coenzyme A and mevalonate in isoprenoid synthesis. There are two classes of HMGR: class I enzymes which are found predominantly in eukaryotes and contain N-terminal membrane regions and class II enzymes which are found primarily in prokaryotes and are soluble as they lack the membrane region. Human (belongs to class I subfamily) and bacterial HMGR (belongs to class II subfamily) differ in their active site architecture (12, 13). Class I HMGRs generally bind to HMG, HMG-CoA in a NADP dependent reaction while class II HMGRs bind to HMG-COA, mevalonate and NAD. Ten sites were selected as specificity determining at which most differences observed in catalytic and substrate binding properties between the class I and II HMGRs (12, 13).

*cd00423:*

This family (Pterin binding enzymes*)* includes dihydropteroate synthase (DHPS) subfamily and cobalamin-dependent methyltransferases. Both DHPS and cobalamin-dependent methyltransferases bind to pterin substrates while sulfonamide drugs act as a specific ligand to DHPS. Four sites that could be important for sulfonamide binding in DHPS (14-16) were considered as specificity determining sites.

*cd00985:*

Maf_Ham1 domain family (cd00985) contains two subfamilies, Ham1 and Maf. A Ham-related protein from *Methanococcus jannaschii* is a novel NTPase that has been shown to hydrolyze nonstandard nucleotides, such as hypoxanthine/xanthine NTP, but not standard nucleotides. Maf, a nucleotide binding protein, has been implicated in inhibition of septum formation in eukaryotes, bacteria and archaea. Three conserved residues could be important for binding to different nucleotides (*e.g*., 2'-Deoxyuridine 5'-Triphosphate (dUTP) and Xanthosine 5'-Triphosphate (XTP) for Maf and Ham1, respectively) and therefore can be regarded as specificity determining sites (17, 18).

*G protein alpha subunit family:*

G_α_ subunits of G protein can be divided into four main subtypes where each of the classes performs different biological functions through specific interactions with the effectors (*e.g*. cyclic GMP phosophodiesterase (PDE)) and regulators (*e.g*. Regulator of G protein signaling (RGS) domains). Multiple sequence alignment were obtained from Pei *et al*., 2006 (19), where the main four subtypes are further divided into 11 subfamilies depending either on the taxonomy (like, plant, animal, fungal G proteins) and type of functions involved (e.g. stimulation G_s_; inhibition G_i_, etc). Sites that were predicted as being specificity determining by both SPEL (19) and SDP-pred (7, 8) methods and were also found to be spatially proximal to the specific effectors or regulators were considered true positives in our analysis.

*Glutathione S-transferase family:*

Glutathione S-transferase (GST) enzymes function to detoxify a wide variety of xenobiotic substrates with reactive electrophilic groups by conjugation to the tri-peptide glutathione (GSH). GST enzymes have been extensively characterized and are grouped according to a robust classification system that is based on a variety of criteria including primary structure, immunoblotting, kinetic properties, inhibitor sensitivity, tertiary structure, and quaternary structure (20).

*LacI/PurR family:*

The LacI/PurR family of transcription factors is regulated by small molecules, such as sugars and nucleotides. In addition to available experimental and structural information, the LacI/PurR family has been widely used by researchers for prediction of specificity determining sites. This family contains 15 specificity groups: AraR, KdgR, CcpA, DegA, YjmH, RbsR, PurR, CytR, GalSR, AscG, LacI, TreR, GntR, IdnR, and FruR. Generally, researchers (7,8,19,21, 22) have identified specificity determining sites through examination of possible contacts between ligand molecules (effector and DNA) and amino acid residues or between amino acid residues of different subunits.

*RICIN domain family:*

A single RICIN domain can be divided into three subunits of approximately 40 amino acids in length that have evolved from an ancient galactose binding peptides (23). The first domain subfamily possesses two carbohydrate binding sites and a peptide binding region (group II), the second domain subfamily contains the N-terminal carbohydrate binding region (group I) and the third one covers the C-terminal carbohydrate binding region (group III) (24).

*Carbohydrate-binding module, family 9:*

The alignment of carbohydrate-binding module family 9 (CBM9) containing approximately 19 putative sequences from 11 organisms was obtained from Notenboom *et al*., 2001 (25). CBM9 family contains two subfamilies: 9a and 9b. Family-9a comprises the N-terminal part of tandem CBM9 modules. The Family-9a module suggests lack of carbohydrate-binding function compared to the other subfamily Family-9b which binds to amorphous and crystalline cellulose and different soluble di- and monosaccharide (25).

*Isocitrate /Isopropylmalate dehydrogenase*

Isocitrate and isopropylmalalte dehydrogenases (IDH and IMDH) are homologous enzymes that oxidize their substrates using NAD or NADP as cofactors. Therefore, these enzymes possess specificities towards the substrate and the cofactor, appearing in four combinations such as, NAD-dependent IDH, NADP-dependent IDH type I, NADP-dependent IDH type II, and NAD-dependent IMDH (26). 14 sites were selected as specificity determining sites which were shown to be in close contact with substrate and/or cofactor by previous computational and experimental studies (26-31).

*Serine protease*

Serine proteases, such as trypsin, chymotrypsin and elastases have similar catalytic mechanisms but have different preferences for the bonds that they preferentially cleave. Trypsins cleave C-terminal to the positively charged amino acid residues, arginine and lysine. Chymotrypsins cleave bond that are flanked by large aromatic residues. Elastases cleave peptide bonds that are next to small-uncharged amino acid residues. These differences in specificity are caused by change of shape of the binding pocket. Aspertate and alanine at positions 189 and 221 are found to be specific for trypsin (32).

*Nucleotidyl cyclase*

Nucleotidyl cyclases catalyse the reaction that transforms a nucleotide triphosphate into a cyclic nucleotide monophosphate. The cyclases act on either guanylate (GUC) or adenylate (ADC). Two point mutations, Glu-Lys and Cys-Asp, are sufficient to change the specificity of the enzyme from GUC to ADC (32-33).

*Lactate/Malate dehydrogenases*

Although lactate/malate dehydrogenases share a similar substrate-binding domain (NAD binding domain) they are found to be highly divergent and as such it is difficult to distinguish between lactate and malate subtypes. A single mutation Gln-Arg (position 102) is known to switch specificity from lactate to malate (32,34-35).

*C and N terminal domain of myc family:*

The *myc* gene family consists of five duplicate protooncogenes (c-myc, L-myc, N-myc, B-myc and s-myc) that encode protein transcription factors for the regulation of cell proliferation and differentiation. The c-*myc* protooncogene is the best-known member of the family and is widely expressed among different tissues in vertebrates. In turn, the N-*myc* genes show much more limited expression. In this study, an alignment comprising 34 proteins from c-myc and N-myc families was used and specificity sites were obtained from Knudsen and Miyamoto, 2001 (36).

*Smad*

The Smad family of transcription factors plays an important role in the transforming growth factor-β signaling pathway and is critical for determining the specificity between alternative pathways (37-40). The family is divided into two major classes: AR-Smads (induced by TGFβ-type receptors) and BR-Smads (induced by the BMP-type receptors). The alignment of 20 nonredundant Smad sequences (Smad-MH2 domain) and the specificity determining residues were collected from Pirovano et al. 2006 (38).

*Rab56 and RasRal*

The Ras superfamily of small GTP-ases is involved in the regulation of growth, survival, differentiation and other processes cells (41). Experimental evidence for functional sites was available from the literature for the Rab 5 versus Rab 6 subfamilies, and the Ras versus Ral families, as discussed in Pirovano et al. 2006 (38). The 28 and 12 specificity sites identified for Rab56 and RasRal, respectively, are listed in Additional file 10.

**Reference:**

1. Santelli E, Richmond TJ. Crystal structure of MEF2A core bound to DNF at 1.5A resolution. *J Mol Biol* 2000; **297**: 437-449.
2. Tan S, Richmond TJ. Crystal structure of the yeast MATalpha2/MCM1/DNF ternary complex. *Nature* 1998; **391**: 660-666.
3. Ooi CE, Weiss J, Elsbach P, Frangione B, Mannion B. A 25-kDa NH2-termiNFl fragment carries all the antibacterial activities of the human neutrophil 60-kDa bactericidal/permeability-increasing protein. *J Biol Chem* 1987; **262**: 14891-14894.
4. Ooi CE, Weiss J, Doerfler ME, Elsbach P. Endotoxin-neutralizing properties of the 25 kD N-terminal fragment and a newly isolated 30 kD C-terminal fragment of the 55-60 kD bactericidal/permeability-increasing protein of human neutrophils. *J Exp Med* 1991; **174**: 649-655.
5. Abrahamson SL, Wu HM, Williams RE, Der K, Ottah N, Little R, Gazzano-Santoro, H, Theofan G, Bauer R, Leigh S, Orme A, Horwitz AH, Carroll SF, Dedrick RL. Biochemical characterization of recombinant fusions of lipopolysaccharide binding protein and bactericidal/permeability-increasing protein. Implications in biological activity. *J Biol Chem* 1997; **272**: 2149-2155.
6. Beamer LJ, Carroll SF, Eisenberg D. Crystal Structure of Human BPI and Two Bound Phospholipids at 2.4 Angstrom Resolution. *Science* 1997; **276**: 1861-1864.
7. Kalinina OV, Novichkov PS, Mironov AA, Gelfand MS, Rakhmaninova AB. SDPpred: a tool for prediction of amino acid residues that determine differences in functional specificity of homologous proteins. *Nucleic Acids Res* 2004; **32**: W424-428.
8. Kalinina OV, Mironov AA, Gelfand MS, Rakhmaninova AB. Automated selection of positions determining functional specificity of proteins by comparative analysis of orthologous groups in protein families. *Protein Sci* 2004; **13**: 443-456.
9. Fu D, Libson A, Miercke LJ, Weitzman C, Nollert P, Krucinski J, Stroud RM. Structure of a glycerol-conducting channel and the basis for its selectivity. *Science* 2000; **290**: 481–486.
10. Sui H, Han BG, Lee JK, Walian P, Jap BK. Structural basis of water-specific transport through the AQP1 water channel. *Nature* 2001; **414**: 872–878.
11. Moor SA, Ronimus RS, Roberson RS, Morgan HW. The Structure of a Pyrophosphate-Dependent Phosphofructokinase from the Lyme Disease Spirochete *Borrelia burgdorfer.* *Structure* 2002; **10**: 659–671.
12. Bochar DA, Stauffacher CV, Rodwell VW. Sequence Comparisons Reveal Two Classes of 3-Hydroxy-3-methylglutaryl Coenzyme A Reductase. *Molecular Genetics and Metabolism* 1999; **66**: 122–127.
13. Istvan ES. Bacterial and mammalian HMG-CoA reductases: related enzymes with distinct architectures. *Curr Opin Struct Biol* 2001; **11**:746-751.
14. Hampele IC, D'Arcy A, Dale GE, Kostrewa D, Nielsen J, Oefner C, Page MG, Schonfeld HJ, Stuber D, Then RL. Structure and function of the dihydropteroate synthase from *Staphylococcus aureus*. *J Mol Biol* 1997; **268**: 21-30.
15. Achari A, Somers DO, Champness JN, Bryant PK, Rosemond J, Stammers DK. Crystal structure of the anti-bacterial sulfonamide drug target dihydropteroate synthase. *Nat Struct Biol* 1997; **4**: 490-497.
16. Doukov T, Seravalli J, Stezowski JJ, Ragsdale SW. Crystal structure of a methyltetrahydrofolate- and corrinoiddependent methyltransferase. *Structure* 2000; **8**: 817–830
17. Minasov G, Teplova M, Stewart GC, Koonin EV, Anderson WF, Egli M. Functional implications from crystal structures of the conserved *Bacillus subtilis* protein Maf with and without dUTP. *Proc Natl Acad Sci U S A* 2000; **97**: 6328–6333.
18. Hwang KY, Chung JH, Kim SH, Han YS, Cho Y. Structure-based identification of a novel NTPase from *Methanococcus jannaschii. Nat Struct Biol* 1999; **6**: 691-696.
19. Pei J, Cai W, Kinch LN, Grishin NV. Prediction of functional specificity determinants from protein sequences using log-likelihood ratios. *Bioinformatics* 2006; **22**: 164-171.
20. Sheehan D, Meade G, Foley VM, Dowd CA. Structure, function and evolution of glutathione transferases: implications for classification of non-mammalian members of an ancient enzyme superfamily. *Biochem J* 2001; **360**: 1-16.
21. Mirny LA, Gelfand MS. Using orthologous and paralogous proteins to identify specificity-determining residues in bacterial transcription factors. *J Mol Biol* 2002; **321**: 7-20.
22. Suckow J, Markiewicz P, Kleina LG, Miller J, Kisters-Woike B, Müller-Hill BGenetic studies of the Lac repressor. XV: 4000 single amino acid substitutions and analysis of the resulting phenotypes on the basis of the protein structure. *J Mol Biol* 1996; **261**: 509-523.
23. Rutenber E, Ready M, Robertus JD. Structure and evolution of ricin B chain. *Nature* 1987; **326**: 624-626.
24. Pils B, Copley RC, Schultz J. Variation in structural location and amino acid conservation of functional sites in protein domain families. *BMC Bioinformatics* 2005; **6**: 210-219.
25. Notenboom V, Boraston AB, Kilburn DG, Rose DR. Crystal Structures of the Family 9 Carbohydrate-Binding Module from *Thermotoga maritima* Xylanase 10A in native and Ligand-Bound Forms. *Biochemistry* 2001; **40**: 6248-6256.
26. Kalinina O.V., Gelfand M.S. Amino Acid Residues that Determine Functional Specificity of NADP- and NAD-Dependent Isocitrate and Isopropylmalate Dehydrogenases. *Proteins.* 2006, **64**:1001–1009.
27. Xu X., Zhao J., Peng B., Huang Q., Arnold E., Ding J. Structure of human cytosolic NADP-dependent dehydrogenase reveals a novel self-regulatory mechanism of activity. *J Biol Chem.* 2004, **279**:22946–33957.
28. Miyazaki K., Yaoi T., Oshima T. Expression, purification, and substrate specificity of isocitrate dehydrogenase from *Thermus* *thermophilus* HB8. *Eur J Biochem*. 1994., **221**:899–903.
29. Doyle S.A., Beernink P.T., Koshland D.E. Jr. Structural basis for a change in substrate specificity: crystal structure of S113E isocitrate dehydrogenase in a complex with isoporpylmalate, Mg2+ and NFDP. *Biochemistry* 2001, **40** :4234–4241.
30. Zhang T. Koshland D.E. Jr. Modeling substrate binding in *Thermus thermophilus* isopropylmalate dehydrogenase. *Protein Sci.* 1995, **4**:84–92.
31. Zhu G., Golding G.B., Dean A.M. The selective cause of an ancient adaptation. *Science* 2005, **307**:1279–1282.
32. Wallace I.M. Higgins D.G. Supervised multivariate analysis of sequence groups to identify specificity determining residues. *BMC Bioinformatics* 2007, **8**:135-146.
33. Tucker CL, Hurley JH, Miller TR, Hurley JB. Two amino acid substitutions convert a guanylyl cyclase, RetGC-1, into an adenylyl cyclase. *Proc Natl Acad Sci U S A*. 1998, **95**: 5993-5997.
34. Wilks HM, Hart KW, Feeney R, Dunn CR, Muirhead H, Chia WN, Barstow DA, Atkinson T, Clarke AR, Holbrook JJ. A specific, highly active malate dehydrogenase by redesign of a lactate dehydrogenase framework. *Science* 1988, **242**:1541-1544.
35. Pazos F, Rausell A, Valencia A. Phylogeny-independent detection of functional residues. *Bioinformatics* 2006, **22**:1440-1448.
36. Knudsen, B & Miyamoto, M.M A likelihood ratio test for evolutionary rate shifts and functional divergence among proteins. *Proc Natl Acad Sci U S A* 2001, **98**: 14512-14517.
37. Ye K, Anton Feenstra K, Heringa J, Ijzerman AP, Marchiori E: Multi-RELIEF: a method to recognize specificity determining residues from multiple sequence alignments using a Machine-Learning approach for feature weighting. *Bioinformatics* 2008, **24**:18-25.
38. Pirovano W, Feenstra KA, Heringa J: Sequence comparison by sequence harmony identifies subtype-specific functional sites. *Nucleic Acids Res*. 2006, **34**: 6540-6548.
39. Feng,X. and Derynck,R. Specificity and versatility in TGF-beta signaling through Smads. *Annu. Rev. Cell Dev. Biol*., 2005, **21**: 659–693.
40. Massagué J, Seoane J, Wotton D. Smad transcription factors. *Genes Dev*. **19**: 2783–2810.
41. Reuther,G. and Der,C. The Ras branch of small GTPases: Ras family members don’t fall far from the tree. *Curr. Opin. Cell Biol*. 2000, **12**: 157–165.
